# A field-based modeling study on ecological characterization of hourly host-seeking behavior and its associated climatic variables in *Aedes albopictus*

**DOI:** 10.1186/s13071-019-3715-1

**Published:** 2019-10-14

**Authors:** Qingqing Yin, Li Li, Xiang Guo, Rangke Wu, Benyun Shi, Yuji Wang, Yingjie Liu, Shang Wu, Yicheng Pan, Qi Wang, Tian Xie, Tian Hu, Dan Xia, Shang Xia, Dzinkambani Moffat Kambalame, Wanyu Li, Zhangyao Song, Siyun Zhou, Ye Deng, Yu Xie, Xiao-Nong Zhou, Chunmei Wang, Xiao-Guang Chen, Xiaohong Zhou

**Affiliations:** 1 Department of Pathogen Biology, Key Laboratory of Prevention and Control for Emerging Infectious Diseases of Guangdong Higher Institutes, Guangdong Provincial Key Laboratory of Tropical Disease Research, School of Public Health, Southern Medical University, Guangzhou, 510515 Guangdong China; 2 WHO Collaborating Centre for Infectious Disease Epidemiology and Control, School of Public Health, Li Ka Shing Faculty of Medicine, The University of Hong Kong, Hong Kong, Hong Kong Special Administrative Region China; 3 The School of Foreign Studies, Southern Medical University, Guangzhou, 510515 Guangdong China; 4 HKBU-NIPD Joint Research Laboratory for Intelligent Disease Surveillance and Control, School of Cyberspace, Hangzhou Dianzi University, Hangzhou, 310018 Zhejiang China; 5 National Institute of Parasitic Diseases, Chinese Center for Diseases Control and Prevention, WHO Collaborating Center for Tropical Diseases, National Center for International Research on Tropical Diseases, Ministry of Science and Technology, Key Laboratory of Parasite and Vector Biology, Ministry of Health, Shanghai, 200025 China

**Keywords:** *Aedes albopictus*, Hourly host-seeking behavior, Climatic variables, Mosquito-borne diseases, Field-based modeling, Female biting behavior

## Abstract

**Background:**

The global spread of mosquito-borne diseases (MBD) has presented increasing challenges to public health. The transmission of MBD is mainly attributable to the biting behaviors of female mosquitoes. However, the ecological pattern of hourly host-seeking behavior in *Aedes albopictus* and its association with climatic variables are still not well understood, especially for a precise requirement for establishing an effective risk prediction system of MBD transmission.

**Methods:**

Mosquito samples and data on mosquito hourly density and site-specific climatic variables, including temperature, relative humidity, illuminance and wind speed, were collected simultaneously in urban outdoor environments in Guangzhou during 2016–2018. Kernel regression models were used to assess the temporal patterns of hourly host-seeking behavior in mosquito populations, and negative binomial regression models in the Bayesian framework were used to investigate the associations of host-seeking behavior with climatic variables.

**Results:**

*Aedes albopictus* was abundant, constituting 82% (5569/6790) of the total collected mosquitoes. Host-seeking behavior in *Ae. albopictus* varied across time and was significantly influenced by climatic variables. The predicted hourly mosquito densities showed non-linear relationships with temperature and illuminance, whereas density increased with relative humidity but generally decreased with wind speed. The range of temperature estimates for female biting was 16.4–37.1 °C, peaking at 26.5 °C (95% credible interval: 25.3–28.1). During the favorable periods, biting behavior of female *Ae. albopictus* was estimated to occur frequently all day long, presenting a bimodal distribution with peaks within 2–3 h around both dawn and dusk (05:00–08:00 h and 16:00–19:00 h). Moreover, a short-term association in hourly density between the females and males was found.

**Conclusions:**

Our field-based modeling study reveals that hourly host-seeking behavior of *Ae. albopictus* exhibits a complex pattern, with hourly variation constrained significantly by climatic variables. These findings lay a foundation for improving MBD risk assessments as well as practical strategies for vector control. For instances of all-day-long frequent female biting during the favorable periods in Guangzhou, effective integrated mosquito control measures must be taken throughout the day and night.

## Background

Mosquito-borne diseases (MBD), including dengue, Zika, chikungunya and yellow fever, have been considered an increasing public health challenge worldwide in concert with the rapid spread of *Aedes aegypti* and *Ae. albopictus* mosquitoes in recent decades [1, 2]. Approximately 390 million annual dengue virus (DENV) infections have been estimated globally [3]. Since the re-emergence of dengue in Foshan, Guangdong, in 1978, dengue outbreaks have been increasing in China, primarily in Guangdong Province [4, 5]. Guangzhou, the capital city of Guangdong Province has a subtropical monsoon climate, with an average annual temperature of 22–23 °C, and an average rainfall of 1983 mm, which are suitable for MBD transmission in the favorable seasons. For example, there were 38,036 dengue cases reported in Guangzhou in 2014, accounting for 80.8% of all cases in the largest dengue outbreak in mainland China since 1990 [6]. *Aedes albopictus* ranks among the top 100 invasive species worldwide and transmits DENV as well as a wide range of arboviruses [7]. The native Asian populations of *Ae. albopictus* have been inferred to split into three main clusters in equatorial Malaysia and tropical Thailand, subtropical south China and temperate Japan [8]. This mosquito species in Guangzhou belongs to the cluster in subtropical south China [8]. It is a vital vector responsible for dengue outbreaks in Guangzhou [6, 8, 9] and a potential vector for Zika virus transmission [10]. Both MBD transmission and mosquito populations vary across seasons in local environments and ecological contexts. In Guangzhou, population densities of *Ae. albopictus* increase and peak during April and November as estimated using multiple sampling methods [Breteau index (BI), container index (CI), route index (RI), ovitrap index (OI) and adult mosquito density index (ADI)] [5, 9, 11–14]. Meanwhile, the indigenous dengue cases in the region increase and peak from August to November, which lag the active duration of population densities of *Ae. albopictus* [5, 6].

The biting behaviors of female mosquitoes are of crucial importance for MBD transmission. Female biting behavior, population size and seasonal dynamics, longevity, dispersal capacity, and vector competence are the key population parameters for mosquito-based surveillance, which provides a basis for further estimations of vectorial capacity and MBD risk (see [15] for a review). The human biting rate (HBR) is an essential indicator of female biting behaviors and is commonly applied in MBD risk forecasting and assessments of vector interventions [16]. Although an in-depth exploration has been performed to find alternative non-exposure and safe mosquito collection methods, the ADI, which is calculated using human landing collection (HLC), remains the standard for providing consistent and reliable HBRs [17]. The human-baited double net trap (HDN) has been verified as a safer alternative to HLC for *Ae. albopictus* monitoring [18, 19]. Thus, the HBRs of wild *Ae. albopictus* populations acquired *via* HDNs or HLC can be utilized for evaluating MBDs and vector control strategies as well as risk forecast modeling such as estimations of the basic reproduction number (*R*_0_) of MBDs transmission under the Ross-Macdonald model framework [20, 21].

The climatic variables associated with hourly host-seeking behaviors in wild populations of *Ae. albopictus* play a key role, especially in establishing a complex forecast model of MBD transmission. Under laboratory conditions, *Ae. albopictus* is verified as a temperature-sensitive mosquito species, with no biting activity occurring below 11 °C or above 36 °C [22]. The survival times of adult *Ae. albopictus* at different temperatures observed in the laboratory could be useful in predicting the dynamics and spread of the population [22]. Although the complicated biogeoclimatic context results in microhabitat diversity in the field, the bioecology, spatiotemporal distribution and vectorial capacity of mosquito vectors have been shown to be constrained by factors such as temperature, relative humidity, wind velocity, light intensity, rainfall, land use, vector control measures and human population density, which in turn affect the spread of MBD [23–26]. With global warming, rapid globalization and urbanization in the past 30 years, dengue transmitted by *Aedes* has been regarded as the fastest spreading arboviral disease [27]. However, the current ecological pattern of hourly host-seeking behaviors of *Ae. albopictus*, together with its association with climatic variables in the outdoor environments, is still not well demonstrated.

In addition, the combination of incompatible and sterile insect techniques (IIT-SIT) provides a promising approach for MBD control, presenting the near elimination of the wild population of *Ae. albopictus* in test islands in Guangzhou [28]. It is accessible for us to illustrate the temporal pattern of hourly activity in wild female and male *Ae. albopictus* populations, which makes it possible to select an appropriate time period for releasing the IIT-SIT males.

Modeling is useful for representing conventional bioecological knowledge in statistical or mathematical frameworks and thus it can be used to identify the influential parameters in MBD transmission and provide a quantitative description [21, 29, 30]. Therefore, we undertook a field-based statistical modeling study to explore the ecological characteristics of hourly host-seeking behavior of *Ae. albopictus* and its associated climatic variables including temperature, relative humidity, illuminance and wind speed in urban outdoor environments in Guangzhou.

## Methods

### Field investigations using HDNs

HDNs (Guangzhou Beikang Biological Vector Monitoring Equipment Company, Guangzhou, China) were selected for the hourly observations in urban outdoor environments in Guangzhou, which included multi-month and multi-site investigations. To explore hourly host-seeking activity patterns of *Ae. albopictus*, and their relationship with site-specific climatic variables, multi-month investigations were designed to account for all weather conditions and lasted for 13 months excluding rainy and/or strong windy days. Meanwhile, hourly observations were conducted in four similar urban outdoor microhabitats during the favorable period with a high mosquito abundance, named multi-site investigations. On each investigation day, hourly observations were performed for 15 min per hour for 24 h [31]. Thereby, a total of 912 hourly observations were recorded, including the multi-month investigations with two repetitions per month at 4–10 days apart (24 h × 2 repetitions × 13 months = 624) from November 2016 to November 2017 and the multi-site investigations with three repetitions per site at 4–10 days apart (24 h × 3 repetitions × 4 sites = 288) during June–July 2018. Mosquito samples and a total of 912 sets of data on mosquito hourly density and site-specific climatic variables including the hourly ambient temperature, relative humidity, illuminance and wind speed were collected simultaneously. The eligible site selection and the process of hourly observation are described in detail in Additional file 1: Text S1.

**Fig. 1:**
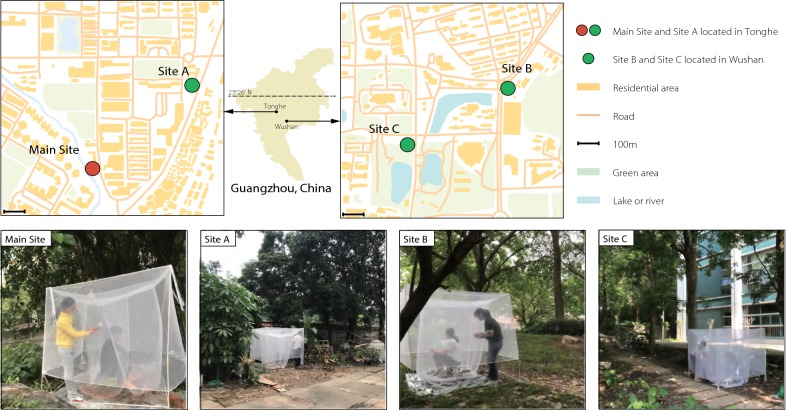
Locations and surrounding outdoor environments of the selected investigation sites in Guangzhou: Main Site (23°11′13″N, 113°19′38″E); Site A (23°11′21″N, 113°19′52″E); Site B (23°9′30″N, 113°21′7″E); and Site C (23°9′26″N, 113°20′59″E). Adobe illustrator CC 2017 with datasets of maps downloaded from Natural Earth (free vector and raster map data at https://www.naturalearthdata.com) was used to generate the maps. Hourly host-seeking densities in wild populations of mosquitoes were observed using the human-baited double net traps (HDNs)

In urban areas of Guangzhou, the site (23°11′13″N, 113°19′38″E), named the Main Site, was selected for the multi-month and multi-site investigations, while another three sites, named Site A (23°11′21″N, 113°19′52″E), Site B (23°9′30″N, 113°21′7″E) and Site C (23°9′26″N, 113°20′59″E), were selected for the multi-site investigations (Fig. 1). Hourly host-seeking activities of adult mosquitoes in the field observed by HDNs were calculated according to the formula: *D* (hourly density of adult mosquitoes, mosquito number per person per hour) =* N* (the total number of adult mosquitoes collected by the 2 collectors in 15 min)/[the 2 collectors × (15 min/60 min per hour)].

### Mosquito species identification

Mosquitoes sampled by the field investigations were frozen at −20 °C for 15 min and morphological species identification was carried out using the taxonomic key by Lu et al. [32]. Further species identification of mosquitoes was confirmed by PCR using the mitochondrial cytochrome *c* oxidase subunit 1 gene (*cox*1) and its sequence analyses (Additional file 2: Text S2).

### Statistical modeling for characterization of hourly host-seeking behaviors in wild *Ae. albopictus* populations

Kernel regression models were used to assess the temporal patterns of the time-series of hourly female and male *Ae. albopictus* densities across time points within a day (TPWADs: 0:00, 1:00, ……, 23:00 h). A bootstrap method was used to estimate the 95% confidence interval of smoothed adult mosquito hourly host-seeking density. Since previous studies reported that a wild population of *Ae. albopictus* from Guangzhou presented significant seasonal dynamics [5, 9, 11–14], we separately examined the temporal patterns in two different periods: the favorable period in November 2016 and from April to November 2017, and the unfavorable period from December 2016 to March 2017.

We explored the association between hourly host-seeking activities of female and male *Ae. albopictus* by examining the association of short-term fluctuations. Considering the consistent temporal patterns of the time-series of female and male mosquito densities across months and across TPWADs could bias their short-term associations, we filtered the time-series of mosquito densities using negative binomial regression models with a categorical variable for the month (*Month*) and TPWAD as the independent variables. Models with an additional random-effect variable-sampling site were used to filter the time-series of adult mosquito densities, which were collected during June-July 2018. Then, we used Spearman’s rank correlation coefficients to assess the associations between the filtered time-series of female and male mosquito hourly densities.

### Statistical modeling for assessing the relationship between climatic variables and hourly host-seeking behaviors in wild *Ae. albopictus* populations

Given that values of mosquito hourly density followed a negative binomial distribution, we applied negative binomial regression models in the Bayesian framework to assess the associations between hourly host-seeking behaviors of mosquitoes and their associated climatic variables, including hourly densities of female and male *Ae. albopictus*, with temperature, relative humidity, illuminance and wind speed. Our aim was to understand the role of potential influential factors on mosquito hourly host-seeking activity in the field, yet sample counts are also influenced by population size. Population size varies on a timescale of months because the generation length of *Ae. albopictus* is approximately 20–30 days [33]. We therefore fitted the models both with and without *Month* as an explanatory variable to help understand the role of potential influential factors. Month probably influenced mosquito densities as well as climatic variables, and thus was likely to be a confounder of their associations. Since a potential confounder should be always considered in the analysis [34], the models with *Month* were then used in our main analysis. The variable of TPWAD was also included into the models. The candidate independent variables (it is not necessary to include all of the candidate independent variables in the model) included temperature (*Temp*), relative humidity (*RH*), illuminance (*Illum*), whether the time point was in the daytime or at nighttime (*d_or_n*) and wind speed (*Wind*). The model for the period between November 2016 and November 2017 is as follows:

$$ \begin{aligned} & \mathop Y\nolimits_{t} \sim NB(r,\;\mathop p\nolimits_{t} ) \hfill \\ & E(\mathop Y\nolimits_{t} ) = \mathop \mu \nolimits_{t} = \frac{{r(1 - \mathop p\nolimits_{t} )}}{{\mathop p\nolimits_{t} }}\;\;\;\;\;\;\;\text{var} (\mathop Y\nolimits_{t} ) = \mathop \mu \nolimits_{t} + \frac{1}{r}\mathop \mu \nolimits_{t}^{2} \hfill \\ & \log (\mathop \mu \nolimits_{t} ) = \alpha + \mathop \beta \nolimits_{TPWAD} \mathop {ns(TPWAD,\;\mathop {df}\nolimits_{TPWAD} )}\nolimits_{t} \\ & \qquad + \mathop \beta \nolimits_{Month} \mathop {Month}\nolimits_{t} \hfill  + \mathop \beta \nolimits_{Temp} \mathop {ns(Temp,\;\mathop {df}\nolimits_{Temp} )}\nolimits_{t}  \\ & \qquad + \mathop \beta \nolimits_{RH} \mathop {ns(RH,\; df = \mathop {df}\nolimits_{RH} )}\nolimits_{t} + \mathop \beta \nolimits_{DN} \mathop {d\_or\_n}\nolimits_{t} \;  \\ & \qquad + \mathop \beta \nolimits_{Wind} \mathop {ns(Wind,\;df = \mathop {df}\nolimits_{Wind} )}\nolimits_{t} \hfill \\ \end{aligned} $$

where $\mathop Y\nolimits_{t}$ and $\mathop \mu \nolimits_{t}$ are the observed and expected mosquito hourly densities at time point *t*, respectively; $r$ and $\mathop p\nolimits_{t}$ are parameters of the negative binomial distribution; *ns* represents the natural cubic spline; *df* stands for degrees of freedom; *Month*_*t*_ indicates the vector of a categorical variable for the month at time point *t*; and *α*, *β*_*ΤPWAD*_, *β*_*Month*_, *β*_*Temp*_, *β*_*RH*_, *β*_*DN*_ and *β*_*Wind*_ are vectors of the regression coefficients for the intercept, TPWAD, *Month*, *Temp*, *RH*, *d_or_n* and *Wind*, respectively.

Since *Temp*, *RH* and *d_or_n* probably mediate the effects of *Illum* on mosquito hourly density, we fitted a separate model that excluded *Temp*, *RH* and *d_or_n* to assess the associations between *Illum* and mosquito hourly densities for the period between November 2016 and November 2017 as follows:

$$ \begin{aligned} \mathop Y\nolimits_{t} \sim NB(r,\mathop p\nolimits_{t} ) \hfill \\ E(\mathop Y\nolimits_{t} ) = \mathop \mu \nolimits_{t} = \frac{{r(1 - \mathop p\nolimits_{t} )}}{{\mathop p\nolimits_{t} }}\;\;\;\;\;\;\;\text{var} (\mathop Y\nolimits_{t} ) = \mathop \mu \nolimits_{t} + \frac{1}{r}\mathop \mu \nolimits_{t}^{2} \hfill \\ \log (\mathop \mu \nolimits_{t} ) = \alpha + \mathop \beta \nolimits_{TPWAD} \mathop {ns(TPWAD,\;\mathop {df}\nolimits_{TPWAD} )}\nolimits_{t} + \mathop \beta \nolimits_{Month} \mathop {Month}\nolimits_{t} \hfill \\ \;\;\;\;\;\;\;\;\;\;\;\;\; + \mathop \beta \nolimits_{Illum} \mathop {ns({ \log }(Illum + 0.001),\;\mathop {df}\nolimits_{Illum} )}\nolimits_{t} + \mathop \beta \nolimits_{Wind} \mathop {ns(Wind,\;df = \mathop {df}\nolimits_{Wind} )}\nolimits_{t} \hfill \\ \end{aligned} $$

where log(*Illum + *0.001) represents the log-transformation of (*Illum* + 0.001) and *β*_*Illum*_ is the corresponding vector of regression coefficients. The clustering effects of sampling sites were considered when fitting models to the data collected during June–July 2018.

The negative binomial regression models were fitted to the data in the Bayesian framework using Markov Chain Monte Carlo methods. We assigned N (0,10^2^) and half-Cauchy (0,5) [35] as the prior distributions for the fixed-effect and random-effect parameters, respectively. We used a 3-chain run for 1000 warm-up and 4000 post-warm-up iterations and kept every fourth iteration, yielding a total of 3000 posterior samples each model. The histograms of the parameter values (excluding those from warm-up iterations) are presented. Trace plots of the values of parameters (excluding those from warm-up iterations) from each chains and potential scale reduction statistic $\hat{R}$ [36] were used to check the convergence of Markov chain Monte Carlo (MCMC) chains.

The *dfs* for the TPWAD, *Temp*, *RH*, *Illum* and *Wind* were selected by the minimum value of the Watanabe-Akaike information criterion (WAIC) [37] for the models. If the WAIC values for two models with different *df*s for one variable were not significantly different, then the most parsimonious model was retained. We included the candidate independent variables one by one in the models. A variable was included into the model if the inclusion of the variable led to a reduction in the WAIC compared with that of the models without the variable. The *R*^2^ for the Bayesian regression models were provided to indicate the proportion of variance in mosquito density explained by the included independent variables [38]. Specifically, we provided the *R*^2^ value for models with TPWAD, *Month*, and one candidate variable (i.e. *Temp*, *RH*, *Illum*, *d_or_n* and *Wind*) and the *R*^2^ for final models with an intercept, TPWAD, *Month* and multiple included variables.

The predicted values of mosquito hourly densities changed with the included independent variables. However, the dose-response curves of mosquito hourly densities and one independent variable for different values of covariates should be parallel. To assess the dose-response relationships between mosquito hourly densities and independent variables, we predicted mosquito hourly densities using all observed values of one included independent variable and setting other independent variables (i.e. covariates) to fixed values. We set the values of continuous covariates to be their medians and TPWAD to be 18:00 h in June. We predicted mosquito hourly densities for the daytime when examining the dose-response curves of mosquito hourly densities and climatic variables (Additional file 3: Table S1). The posterior distributions of parameters were used to estimate the 95% credible intervals (CrI) for the predicted mosquito hourly densities.

To explore the possible ranges of temperatures at which adult mosquitoes exhibit biting behavior, we estimated the ranges of temperatures that corresponded to the predicted adult mosquito densities which were ≥ 1 per person per hour for combinations of different values of independent variables. Specifically, we created all possible combinations of the observed values of climatic variables each month because the observed climatic variables varied across months. Then, we repeated the created combinations each TPWAD, thus forming a design matrix, and thereafter predicted densities of adult mosquitoes for the design matrix. The estimated ranges of temperatures that corresponded to ≥ 1 predicted densities of adult mosquitoes are presented by month.

Sensitivity analyses were conducted to (i) compare results of the correlation between mosquito hourly densities and climatic variables based on the models with and without *Month*; (ii) examine the dose-response curves of mosquito hourly densities and climatic variables, including *Temp*, *RH* and *Wind* based on the models with *Illum* instead of *d_or_n*; and (iii) evaluate the associations between mosquito hourly densities and *Illum* based on the models with variables including *Temp* and *RH*.

All analyses were conducted using R v.3.4.3 (R Foundation for Statistical Computing, Vienna, Austria).

## Results

**Fig. 2:**
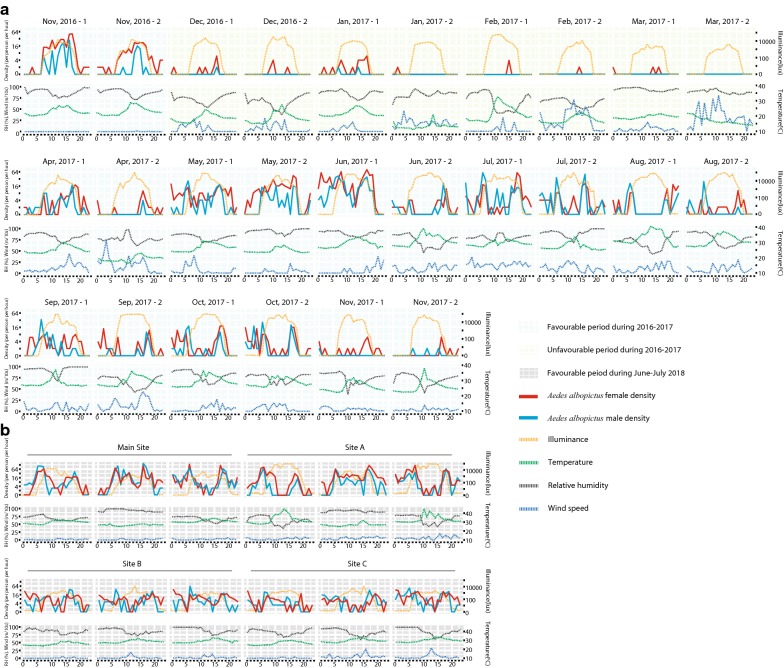
Temporal variations in hourly densities of *Aedes albopictus* and site-specific climatic variables from field investigations. **a** Two repetitions per month at the Main Site from November 2016 to November 2017. **b** The multi-site investigations with three repetitions during June–July 2018. Host-seeking densities of male (blue) and female (red) *Ae. albopictus*, temperature (green), illuminance (orange), relative humidity (black) and wind speed (navy blue) were recorded hourly

A total of 2852 adult mosquitoes were collected during our multi-month investigations from November 2016 to November 2017 (Additional file 4: Table S2), and two species were identified, *Ae. albopictus* and *Cx. quinquefasciatus*. The majority (71%, 2025/2852) of the collected mosquitoes were *Ae. albopictus*, while 29% (827/2852) were *Cx. quinquefasciatus* (Additional file 4: Table S2). Meanwhile, in the multi-site investigations during June-July 2018, we collected 3938 adult mosquitoes. *Aedes albopictus*, *Cx. quinquefasciatus* and *Armigeres subalbatus* (Additional file 5: Table S3) were identified; *Ae. albopictus* with a total of 3544 also accounted for the largest proportion. Consequently, constituting 82% (5569/6790) of the total collected mosquitoes, *Ae. albopictus* presented abundant in outdoor urban environments in Guangzhou, while *Cx. quinquefasciatus* was another common mosquito species, accounting for 17.6% (1193/6790), which is consistent with previous reports [9, 39, 40]. The total numbers of all collected mosquitoes during daytime and nighttime were, respectively, 2300 and 929 females and 1807 and 533 males of *Ae. albopictus*, and 160 and 819 females and 80 and 134 males of *Cx. quinquefasciatus.* In accordance with previous studies, the hourly host-seeking behavior of *Cx. quinquefasciatus* showed a dominant nocturnal pattern (Additional file 4: Table S2, Additional file 5: Table S3). Further statistical modeling of *Cx. quinquefasciatus* was limited by its lower number of collected samples using HDNs in this study. Although the predicted hourly densities of female and male *Ae. albopictus* were still observed to be higher during the daytime than during the nighttime [33] (Fig. 2, Additional file 4: Table S2, Additional file 5: Table S3, Additional file 6: Table S4), our field-based modeling indicated that hourly host-seeking behavior of *Ae. albopictus* exhibited a complex pattern as the following demonstration shows in detail.

### Ecological characteristics of hourly host-seeking behaviors in *Ae. albopictus* revealed by the field-based modeling study

**Fig. 3:**
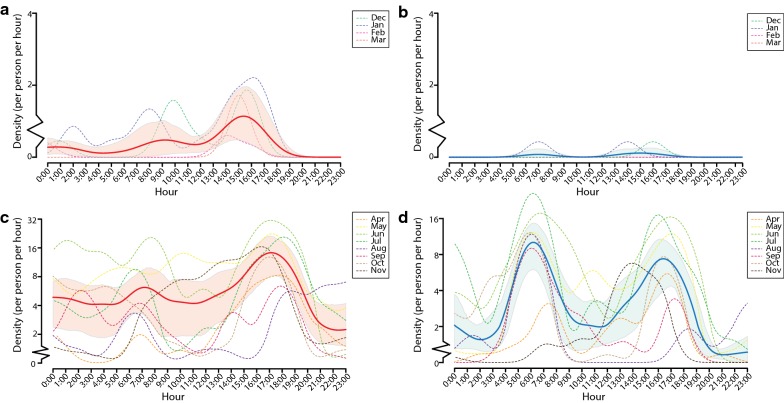
Temporal patterns in hourly host-seeking activities of *Aedes albopictus* from multi-month investigations. **a**–**d** Hourly dynamics of host-seeking behaviors found in female (**a**) and male (**b**) *Ae. albopictus* from multi-month investigations during the unfavorable period in 2016–2017 and female (**c**) and male (**d**) *Ae. albopictus* from multi-month investigations during the favorable period in 2016–2017. The smoothed hourly densities of female and male *Ae. albopictus* are displayed in solid red and blue lines, respectively. The shaded areas represent 95% credible intervals of the smoothed hourly densities

**Fig. 4:**
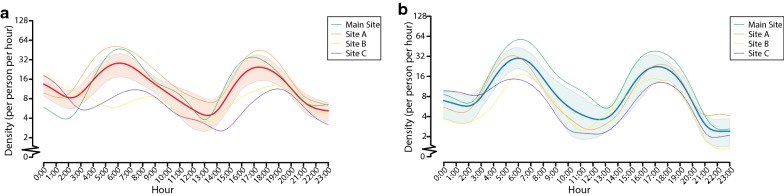
Temporal patterns in hourly host-seeking activities of *Aedes albopictus* from multi-site investigations. **a**, **b** Hourly dynamics of host-seeking behaviors found in female (**a**) and male (**b**) *Ae. albopictus* from multi-site investigations during June–July 2018. The smoothed hourly densities of female and male *Ae. albopictus* are displayed in solid red and blue lines, respectively. The shaded areas represent 95% credible intervals of the smoothed hourly densities

In the present study, hourly host-seeking behaviors of *Ae. albopictus* represented a complex pattern with significant variations on an hourly basis (Figs. 3, 4) and generally varied across months and sites.

In the multi-month investigations, hourly densities ranged between 0–78 (mean of 4) and 0–58 (mean of 2) for female and male *Ae. albopictus*, respectively (Figs. 2a, 3). Consistent with the seasonal variations in population densities of *Ae. albopictus*, as well as dengue morbidity in Guangzhou reported in previous studies [5, 6, 9, 11–14], hourly host-seeking activities of *Ae. albopictus* also presented a seasonal variation (Additional file 7: Figure S1), lower during the unfavorable period from December 2016 to March 2017 (Figs. 2a, 3a, b), but higher during the favorable period in November 2016 and April to November 2017, especially from May to July 2017 (Figs. 2a, 3c, d).

During the favorable period with high mosquito abundance from June to July 2018, the multi-site investigations further demonstrated that hourly densities were higher, ranging between 0–138 (mean of 13) and 0–156 (mean of 11) for female and male *Ae. albopictus*, respectively (Figs. 2b, 4). Although the four sites were carefully selected based on the same selection criteria, heterogeneous hourly activities of *Ae. albopictus* were observed between Tonghe (Main Site and Site A, 23°11′N, 113°19′E) and Wushan (Site B 23°9′N, 113°21′E and Site C 23°9′N, 113°20′E) (Figs. 2b, 4).

*Aedes albopictus* is commonly known as a predominantly diurnal biting species [41], but we found that host-seeking activities of *Ae. albopictus* occurred frequently all day long during the favorable period through both the multi-month and multi-site field-based modeling analyses (Figs. 2, 3, 4). In the multi-month investigations, the highest hourly biting densities of females during the day and night were 78 and 58, respectively, occurring in June 2017. Meanwhile, in the multi-site investigations, the highest female hourly biting densities reached 138 and 78 during the daytime and nighttime, respectively.

In the multi-month investigations, the peak period of smoothed hourly densities of female *Ae. albopictus* occurred between 16:00 and 18:00 h, and a small peak occurred between 6:00 and 8:00 h from April to November (Fig. 3c). During the unfavorable period from December 2016 to March 2017, the peak time was from 14:00 to 16:00 h (Fig. 3a). Hourly host-seeking activities over 24 hours were also analyzed in the male mosquito populations (Fig. 3b, d). Double peaks were observed in the smoothed hourly densities of male, with the first peak occurring between 05:00 and 08:00 h and the second peak occurring between 16:00 and 18:00 h in the favorable period (Fig. 3d). Meanwhile, in the multi-site investigations, the smoothed hourly density of females peaked between 05:00 and 08:00 h and between 16:00 and 19:00 h, with variations among the four sites (Figs. 2b, 4a). Double peaks were also observed in the smoothed hourly densities of males, with the first peak occurring between 05:00 and 07:00 h and the second peak occurring between 16:00 and 18:00 h (Figs. 2b, 4b).

Interestingly, we found a short-term association between the hourly activity of female and male mosquitoes by both the multi-month and multi-site modeling analyses (between November 2016 and November 2017: *r*_*s*_ = 0. 545, *N* = 624, *P* < 0.001; June and July 2018: *r*_*s*_ = 0. 470, *N* = 288, *P* < 0.001).

### Association analyses between hourly host-seeking activity of adult *Ae. albopictus* and climatic variables

In the multi-month investigations, the site-specific temperature, relative humidity, illuminance and wind speed ranged between 11.1–40.4 °C, 37–99%, 0–116,000 lux and 0–7.9 m/s, respectively, with means of 24.7 ± 5.8 °C, 79.5 ± 13.5%, 10,115.71 ± 22,288.20 lux and 0.9 ± 1.2 m/s, respectively (Fig. 2a). Two final models for females were constructed for the period between November 2016 and November 2017: (i) a model with an intercept, TPWAD, *Month*, *Temp*, *RH*, *d_or_n* and *Wind* (Additional file 8: Table S5a); and (ii) a model with an intercept, TPWAD, *Month*, *Illum* and *Wind* (Additional file 8: Table S5b). The independent variables of the two final models for females respectively explained 45.6% (95% CrI: 34.7–54.5%) and 46.3% (95% CrI: 35.0–54.2%) of the variance in hourly densities of females. The two final models for males were fitted for the period between November 2016 and November 2017: (i) a model with an intercept, TPWAD, *Month*, *Temp*, *RH* and *d_or_n* (Additional file 8: Table S5c); and (ii) a model with an intercept, TPWAD, *Month*, and *Illum* (Additional file 8: Table S5d). The included independent variables of the two final models for males respectively explained 58.5% (95% CrI: 44.2–64.2%) and 51.5% (95% CrI: 35.4–58.5%) of the variance in hourly densities of males. The point estimate of *R*^2^ for the model with *Illum* for females was higher than those for the models with other variables, but the 95% CrI of the *R*^2^ values for models with different variables overlapped (Additional file 9: Table S6).

The temperature, relative humidity, illuminance and wind speed, which were recorded in the multi-site investigations, ranged between 23.6–45.1 °C, 37.0–99.0%, 0.0–91,200.0 lux and 0.0–3.3 m/s, respectively, with means of 29.3 ± 3.3 °C, 80.6 ± 13.8%, 4383.83 ± 15,583.14 lux and 0.3 ± 0.4 m/s, respectively (Fig. 2b). The mean temperature and illuminance were higher at the Main Site and Site A than at Site B and Site C. Two final models were constructed for females and males during June–July 2018: (i) a model with an intercept TPWAD, *Month*, *Temp* and *d_or_n*, (Additional file 10: Table S7a, b); and (ii) a model with an intercept TPWAD, *Month* and *Illum* (Additional file 10: Table S7c, d). The included independent variables of the two final models for females respectively explained 19.2% (95% CrI: 1.4–50.8%) and 5% (95% CrI: 0.3–42.8%) of the variance in hourly densities of females, and the included independent variables of the two final models for male respectively explained 29.4% (95% CrI: 1.9–50.5%) and 7.7% (95% CrI: 0.5–38.3%) of the variance in hourly densities of males.

**Fig. 5:**
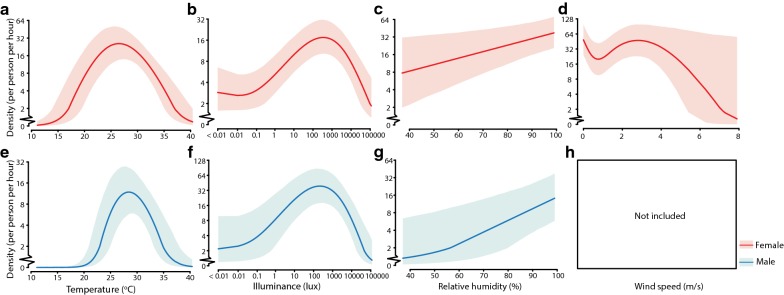
Dose-response relationships between hourly host-seeking activities of *Aedes albopictus* and climatic variables from multi-month investigations. The solid lines represent the predicted values of *Ae. albopictus* hourly densities by temperature, illuminance, relative humidity and wind speed, assuming that the other continuous covariates were equal to their medians and that the time point was at 18:00 h in June. The shaded areas represent the 95% credible intervals of the predicted hourly densities. Dose-response relationships between hourly host-seeking activities of female (**a**–**d**), male (**e**–**h**) *Ae. albopictus* and temperature, illuminance, relative humidity and wind speed in multi-month investigations from November 2016 to November 2017

In the multi-month investigations, the dose-response curves of the mosquito hourly densities and independent variables are presented in Fig. 5. The predicted hourly female and male densities initially increased with temperature, peaking at 26.5 °C (95% CrI: 25.3–28.1) and 28.4 °C (95% CrI: 27.0–30.4), respectively, and then decreased with temperature (Fig. 5a, e). Similarly, the predicted hourly female and male densities also initially increased with illuminance, peaking at 348.20 lux (95% CrI: 136.49–825.81) and 221.49 lux (95% CrI: 68.74–555.72), respectively, and then decreased with illuminance (Fig. 5b, f). Meanwhile, the predicted hourly female and male densities increased with increasing relative humidity and generally decreased with increasing wind speed (Fig. 5c, d, g).

**Fig. 6:**
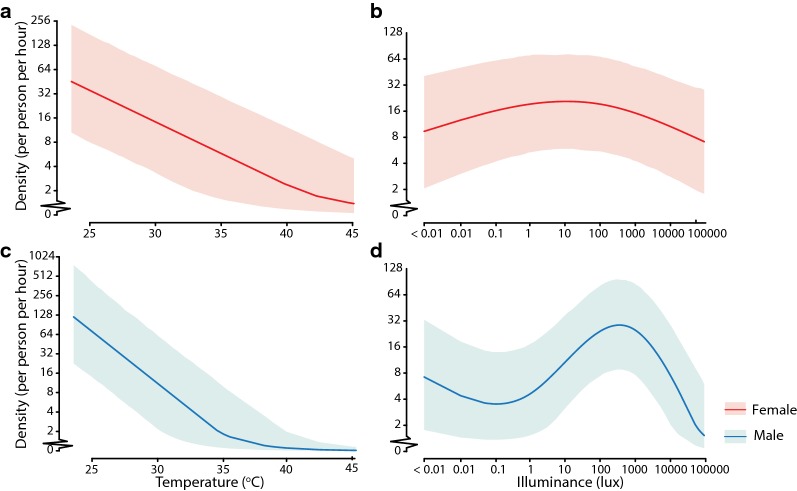
Dose-response relationships between hourly host-seeking activities of *Aedes albopictus* and temperature, illuminance from multi-site investigations. The solid lines represent the predicted values of *Ae. albopictus* hourly densities by temperature and illuminance, assuming that the other continuous covariates were equal to their medians and that the time point was at 18:00 h in June. Dose-response relationships between hourly host-seeking activities of female (**a**, **b**), male (**c**, **d**) *Ae. albopictus* and temperature and illuminance in multi-site investigations during June–July 2018

Similar associations of adult mosquito hourly density with independent climatic variables were further verified by the multi-site investigations (Fig. 6). During June-July 2018, the predicted hourly female and male densities initially peaked and then decreased with temperature fluctuation (Fig. 6a, c). Meanwhile, the predicted hourly female and male densities also initially increased with illuminance, peaking at 11.93 lux (95% CrI: 0.35–187.17) and 369.15 lux (95% CrI: 179.59–602.72), respectively, and then decreased with illuminance (Fig. 6b, d). Notably, relative humidity and wind speed were not included in the models for the multi-site investigations because the inclusion of these variables would not lead to a reduction in the WAIC. Trace plots of the parameter values suggested that the MCMC chains have attained stationarity and mixed, which indicated the convergence of MCMC chains (Additional file 11: Figure S2). $\hat{R}\; < \;1.1$ also suggested that the MCMC chains were converged.

The estimated range of temperatures corresponding to predicted hourly densities ≥ 1 per person per hour for *Ae. albopictus* found by the multi-month modeling are presented in Additional file 12: Table S8. The minimum and maximum temperatures that corresponded to a predicted female biting density ≥ 1 per person per hour were 16.4 and 37.1 °C, respectively, whereas those that corresponded to a predicted male density ≥ 1 per person per hour were 21.7 and 37.1 °C, respectively.

The sensitivity analyses indicated that (i) month was likely to be a confounder of the associations between mosquito densities and climatic variables given the differences observed in their estimated associations using the models with and without *Month* (Figs. 5, 6 and Additional file 13: Text S3a, b); (ii) the dose-response curves of mosquito hourly densities and climatic variables, including *Temp*, *RH* and *Wind* estimated from the models with *Illum* but without *d_or_n* were similar to those in the main analysis (Additional file 13: Text S3c, d); and (iii) *Temp* and *RH* could mediate the effects of *Illum* on mosquito hourly densities given the differences observed between the models with and without *Temp* and *RH* (Additional file 13: Text S3e).

## Discussion

In the present field-based modeling, the ecological characteristics of hourly host-seeking behaviors of *Ae. albopictus* and the associated site-specific climatic variables including temperature, illuminance, relative humidity and wind speed were systematically clarified. The results demonstrated that hourly host-seeking behaviors of *Ae. albopictus* present a complex pattern with an hourly variation and are significantly influenced by climatic variables.

The seasonal fluctuations in the population densities of *Ae. albopictus* and dengue morbidity have been recorded in Guangzhou [5, 6, 9, 11–14]. The divergence of host-seeking behavior in *Ae. albopictus* across site-specific microhabitats was also described in earlier observations [33, 42]. Similar to these previous studies, the heterogeneity and complexity of host-seeking behavior of *Ae. albopictus* were confirmed across sites (Figs. 2b, 4) and months (Figs. 2a, 3). For instance, divergence across places was found from Tonghe to Wushan in our multi-site investigations. The hourly variation in host-seeking behavior of *Ae. albopictus* was likely attributable to all variances of the influential factors including time points, microhabitats and the inherent bioecological characteristics of *Ae. albopictus* and was crucially constrained by climate variances.

### Hourly variation in host-seeking behavior of *Ae. albopictus* exhibited bimodality and female biting occurred frequently all day long during the favorable period

As is known, *Ae. albopictus* usually bites during daytime [41]. However, during the favorable period both in the multi-month and multi-site investigations in urban outdoor environments, we found that hourly host-seeking activities of adult *Ae. albopictus* occurred frequently all day long, and that females in particular presented aggressive biting behavior throughout the day and night. Although the hourly densities were higher during the day, female biting also occurred frequently at night. During June-July 2018, the female hourly biting density reached up to 78 at night. *Aedes albopictus* is strongly anthropophilic and has a higher blood-feeding rate in urban areas, where the human population density is greater [43]. The environmental changes caused by urbanization have had a considerable impact on the ecology of *Ae. albopictus* [44]. The more frequent nighttime biting of female *Ae. albopictus* in urban outdoor environments in Guangzhou during the favorable periods may be attributed to complex factors, including increasing human population density, more larval breeding habitats, bright city lights and changing climatic conditions due to global warming. The all-day-long biting activity of females may pose an increasing risk of dengue transmission. Therefore, effective measures must be taken to prevent the biting activity of *Ae. albopictus* throughout both the day and night.

In addition, previous observations also discovered a bimodal pattern of female *Ae. albopictus* biting: (i) one peak at dawn and one peak occurring in the afternoon, with lower nonzero activity levels between these two peaks in Macao, China [45]; (ii) the highest host-seeking activity peak occurring approximately two hours before sunset and a smaller but important activity peak occurring at 8:30 h on La Réunion Island in the Indian Ocean [46]. Documented in a monograph on dengue fever and its vectors in China [33], field investigations focusing on the biting behavior of *Ae. albopictus* had been launched in Fuzhou, Fujian Province (Wang, 1952), Shanghai (Liu, 1958), and six sites in Yixing, Jiangsu Province; Nanyang and Boai, Henan Province; and Nanning, Guangxi Province (the National Cooperation Group, China, 1979). Most of the hourly observations using HDNs were performed in outdoor environments, especially in bamboo forest microhabitats with abundant mosquitoes during 04:00–20:00 h or all-day-long. A descriptive analysis of these data indicated that patterns of biting behaviors in the wild populations of *Ae. albopictus* were similar in different areas in China, indicating this species is a dominant diurnal biter with two biting peaks, the first at one to two hours around the local sunrise and the other at two to three hours before the local sunset, with the latter peak being higher.

In agreement with these previous studies, our field-based modeling provided an in-depth quantitative description of the bimodality in hourly host-seeking behavior of *Ae. albopictus*. During the favorable period, the highest female biting activity occurred from 16:00 to 18:00 h based on the modeling estimates from the multi-month investigations. Meanwhile, the multi-site investigations estimated that female biting primarily peaked from 16:00 to 19:00 h. The secondary peaks of female biting occurred from 06:00 to 08:00 h and 05:00 to 08:00 h, estimated by the multi-month and multi-site field-based modeling, respectively. Although it varies slightly depending on different times and locations, the bimodality of female biting behavior is similar to that described previously [45, 46]. Thus, the bimodality of female biting could be recognized as appearing within two to three hours around both dawn and dusk, i.e. 05:00–08:00 h and 16:00–19:00 h, in urban outdoor environments in Guangzhou during the favorable period. Therefore, we also call for greater awareness of the high risk of female biting, as well as dengue transmission during the peak times, among governments, health authorities and the general public.

### Hourly host-seeking behaviors of *Ae. albopictus* are significantly influenced by climatic variables

In our study, field-based negative binomial regression models in the Bayesian framework were employed to identify the associations between hourly host-seeking behaviors of *Ae. albopictus* and climatic variables. Since month could be a potential confounder of their associations, *Month* was then included in the main models. We focused on the effects of climatic variables on mosquito activity instead of its population size. The variable patterns of host-seeking behaviors of *Ae. albopictus* in urban outdoor environments in Guangzhou were found to be significantly influenced by climatic variables.

Previous studies reported that climatic variables, including temperature, rainfall, etc., influenced the spatiotemporal distributions and dynamics of vector populations as well as the transmission of vector-borne diseases [23–25, 33, 47, 48]. Among these factors, temperature plays a crucial role in the establishment of a population, mosquito dynamics and dengue transmission mediated by *Ae. albopictus* [49–53]. Shen et al. [54] reported that a minimum monthly temperature threshold of 18.25 °C might have been associated with the dengue incidence from June to November during 2006–2014 in Guangzhou. Xiao et al. [55] estimated in a laboratory study that no transmission might occur below 18 °C based on their findings from *Ae. albopictus* infected orally with a DENV-2 suspension and incubated within a temperature range of 18–36 °C. A non-linear relationship was also found between temperature and dengue incidence [56]. A systematic review and meta-analysis indicated that the minimum temperature (18.1–24.2 °C) and maximum temperature (28.0–34.5 °C) were associated with increased dengue transmission in tropical or subtropical areas [57]. Duan et al. [22] observed that no biting activity occurred below 11 °C or above 36 °C under laboratory conditions in *Ae. albopictus* collected from habitats in Jiangsu Province, China. In comparison with the temperature thresholds for dengue transmission or female biting of *Ae. albopictus* described in the previous studies, our field-based modeling further demonstrated that the predicted temperature range suitable for female *Ae. albopictus* biting in urban outdoor environments in Guangzhou was 16.4 to 37.1 °C, with an estimated peak at temperatures of 26.5 °C (95% CrI: 25.3–28.1). The differences in the temperature threshold estimates can be attributed to the different methodologies as well as the complex interactions of environmental factors in outdoor microhabitats.

A previous study reported that the threshold of light intensity for the activation of nocturnal host-seeking activity was greater than 10 lux in *Ae. albopictus* [25]. Interestingly, our field-based modeling found that female *Ae. albopictus* biting peaked under a lower illumination of 11.93 lux (95% CrI: 0.35–187.17) and 348.20 lux (95% CrI: 136.49–825.81) as estimated by our multi-site and multi-month studies, respectively, and both estimates were close to the light densities from dawn and dusk, which might explain the female biting bimodality that appeared within respective two to three hours around dawn and dusk.

### Short-term association of hourly host-seeking activities between female and male *Ae. albopictus*

In our study, more female than male mosquitoes were collected, which is consistent with previous observations using HDNs in Madagascar [58]. Double peaks were also observed in the smoothed values for male density by the kernel regression model, with the first peak occurring between 5:00 and 7:00 or 8:00 h, and the second peak occurring between 16:00 and 18:00 h during the favorable periods in 2016 to 2018. Hourly host-seeking activity patterns of female and male mosquitoes may be related to their mating behavior. Several studies have shown that the mating patterns of *Ae. albopictus* were bimodal and diurnal in Macao, China, Samui Island, Thailand and La Reunión Island [45, 46, 59, 60], and the peaks were recorded during 6:00–8:00 h and 16:00–17:00 h in Macao [45]. Previous studies have reported that *Ae. aegypti* and *Ae. diantaeus* males were waiting for blood-sucking females in the vicinity of the hosts [61, 62]. We found a short-term association between female and male *Ae. albopictus* hourly densities, which suggests that the mating behavior of *Ae. albopictus* in urban outdoor environments in Guangzhou may be associated with female biting. These findings should facilitate improvements to the release strategies of IIT-SIT *Ae. albopictus* males for vectors and MBDs control [28].

### Limitations

In this field-based modeling study, multi-month investigations included two repetitions each month and multi-site investigations for three repetitions each site. We performed 24 hourly observations each investigation day using HDNs, with a total of 912 hourly observations performed and 912 site-specific datasets collected. Although the present field-based modeling study is informational, a further investigation launched in more collection sites for more years may further strengthen the study. In another aspect, we did not estimate the relative importance of each climatic variable even though we researched in the associations between mosquito hourly densities and various climatic variables using negative binomial regression models in the Bayesian framework. Further exploration is needed.

## Conclusions

The present field-based modeling indicated that host-seeking behaviors of *Ae. albopictus* exhibit a complex pattern with hourly variation. Hourly mosquito densities of *Ae. albobictus* had a non-linear relationship with temperature and illuminance. At the same time, the densities increased with relative humidity while decreasing with wind speed. During the favorable period in urban outdoor environments in Guangzhou, female *Ae. albopictus* were shown to bite frequently all day long, with bimodality occurring within two to three hours of both dawn and dusk. These findings lay a foundation for improving MBD risk assessments as well as practical strategies for vector control.

## Supplementary information

**Additional file 1: Text S1.** The eligible site selection and the process of hourly observation.

**Additional file 2: Text S2.** Species identification of the collected adult mosquitoes.

**Additional file 3: Table S1.** The fixed values of other variables.

**Additional file 4: Table S2.** Adult mosquitoes collected by multi-month investigations from November 2016 to November 2017.

**Additional file 5: Table S3.** Adult mosquitoes collected by multi-site investigations during June-July 2018.

**Additional file 6: Table S4.** The predicted values for the multi-month and multi-site mosquito densities of *Ae. albopictus* during both the day and night.

**Additional file 7: Figure S1.** Seasonal variations of hourly host-seeking activities found in *Ae. albopictus* during 2016–2017. **a**, **b** Seasonal variations of hourly host-seeking activities found in female (**a**) and male (**b**) *Ae. albopictus*. The smoothed hourly densities of female and male *Ae. albopictus* are displayed in solid red and blue lines, respectively. The shaded areas represent 95% credible intervals of the smoothed hourly densities.

**Additional file 8: Table S5.** Values of the Watanabe-Akaike information criterion (WAIC) for models used for the assessment of potential influential factors of *Ae. albopictus* for the period from November 2016 to November 2017.

**Additional file 9: Table S6.** *R*^2^ values for the models with time points within a day and month and different variables.

**Additional file 10: Table S7.** Values of the Watanabe-Akaike information criterion (WAIC) for models used for the assessment of potential influential factors of *Ae. albopictus* for the period June-July 2018.

**Additional file 11: Figure S2.** The histograms and trace plots of the parameter values.

**Additional file 12: Table S8.** Estimated thresholds of temperatures that corresponded to the predicted *Ae. albopictus* densities which were ≥ 1 per person per hour from multi-month investigations from November 2016 to November 2017.

**Additional file 13: Text S3.** Sensitivity analyses.

## Data Availability

Data supporting the conclusions of this article are included within the article and its additional files. The datasets used and/or analyzed during the present study are available from the corresponding author upon reasonable request.

## Abbreviations

MBD: mosquito-borne disease
DENV: dengue virus
HBR: human biting rate
BI: Breteau index
CI: container index
RI: route index
OI: ovitrap index
*cox*1: cytochrome *c* oxidase subunit 1 gene
ADI: adult mosquito density index
HLC: human landing catch
HDN: human-baited double net trap
IIT-SIT: the combination of incompatible and sterile insect techniques
TPWAD: time points within a day
MCMC: Markov chain Monte Carlo
WAIC: Watanabe-Akaike information criterion
CrI: credible interval
