# Supplementary material for: A field-based modeling study on ecological characterization of hourly host-seeking behavior and its associated climatic variables in Aedes albopictus
Source: Parasit Vectors. 2019 Oct 14;12:474. doi: 10.1186/s13071-019-3715-1 (PMC6791010; doi:10.1186/s13071-019-3715-1)
Supplement: Supplementary file 1 — Additional file 1: Text S1. The eligible site selection and the process of hourly observation. [file 13071_2019_3715_MOESM1_ESM.pdf]

The eligible site selection and the process of hourly observation

We conducted pilot studies in at least three urban areas of Guangzhou to identify an area with suitable mosquito abundance for hourly sampling as the investigation site. Then, the site in Tonghe (23°11'13"N, 113°19'38"E) was chosen for the multi-month and multi-site investigations, named the Main Site. Tonghe has a population density of > 3,000 people/km^2 and is located in the Baiyun District, the only district of Guangzhou where more than ten thousand dengue cases were documented during the 2014 dengue outbreak [1]. The study site is covered with trees and grass, with a ditch (the larval breeding habitats for Culex spp.) nearby, and it is surrounded by residential buildings, a university and a number of commercial blocks. There are abandoned foam boxes and plastic buckets filled with water presenting larval breeding habitats for Aedes spp. in the site.

In addition to the Main Site, another three sites in urban areas in Guangzhou named Site A (23°11'21"N, 113°19'52"E), Site B (23°9'30"N, 113°21'7"E) and Site C (23°9'26"N, 113°20'59"E), were selected based on the same selection criteria for the multi-site investigations: Site A was at least 500 m away from the Main Site in Tonghe, while Sites B and C were separated by at least 500m in Wushan, Tianhe District. The geographic maps representing the investigation sites which was generated using Adobe illustrator CC 2017 with datasets of maps downloaded from Natural Earth (free vector and raster map data @ naturalearthdata.com.).

The HDN includes internal and external nets and is 1.8 m long×1.8 m wide×1.8 m high. The internal net remains closed, and a distance of 0.3 m is maintained from the external net to the ground while the trap is in operation. The nets were not treated with any insecticide. To catch mosquitoes with the HDN, two collectors occupied the trap, which was not exposed to direct sunlight. One collector occupied the internal net to bait adult mosquitoes into the external net. The other collector occupied the external net and collected the mosquitoes that entered the external net using an electric aspirator mosquito catcher (Guangzhou Plant Protection Insecticidal Services Ltd, Guangzhou, China).

Gao et al. (2018) reported no significant variation in *Ae. albopictus* catching by HDNs between different collectors [2]. In the present study, 12 collectors, including males and females between 20 and 26 years old, were trained and divided into six groups of two. In each group, two collectors operated HDN as described above and collected mosquito specimens and data. The six groups worked for six 4-hour periods from 0:00–

4:00 h, 4:00–8:00 h, 8:00–12:00 h, 12:00–16:00 h, 16:00–20:00 h and 20:00–24:00 h, and rotated time periods after every investigation day. The hourly ambient temperature, relative humidity, illuminance and wind speed in each site were recorded simultaneously by a temperature and humidity meter (Thermo Electron Corporation, Boston, USA), luminometer (Shenzhen Huayi Peakmeter Technology Co. Ltd, Shenzhen, China) and anemometer (Jiaxing Guohao Teaching Instrument Ltd, Jiaxing, China). The times of sunrise and sunset were also recorded each investigation day.

References

1. Cao WQ, Ye HF, Ye SL, Cheng XO, Pang ZM. Epidemiology of dengue fever in Baiyun district of Guangzhou in 2014. J Trop Med. 2016; 16: 371–3.

2. Gao Q, Wang F, Lv X, Cao H, Zhou JJ, Su F, et al. Comparison of the human-baited double net trap with the human landing catch for *Aedes albopictus* monitoring in Shanghai, China. Parasit Vectors. 2018; 11: 483.
