# Supplementary material for: A field-based modeling study on ecological characterization of hourly host-seeking behavior and its associated climatic variables in Aedes albopictus
Source: Parasit Vectors. 2019 Oct 14;12:474. doi: 10.1186/s13071-019-3715-1 (PMC6791010; doi:10.1186/s13071-019-3715-1)
Supplement: Supplementary file 2 — Additional file 2: Text S2. Species identification of the collected adult mosquitoes. [file 13071_2019_3715_MOESM2_ESM.pdf]

Species identification of the collected adult mosquitoes

Methods

Mosquitoes sampled by the field investigation were frozen at -20°C for 15 min and morphological species identification was carried out using the taxonomic key by Lu et al [1]. Further species identification of mosquitoes were confirmed by PCR using mitochondrial cytochrome *c* oxidase subunit 1 gene (*cox*1) and *cox*1 sequence analyses. PCR identifications were done on individually specimens. One each mosquito species per day was selected for molecular identification. A total of 38 *Aedes albopictus*, 38 *Culex quinquefasciatus* and 2 *Armigeres subalbatus* were selected. Total DNA was extracted from individual adult mosquitoe using the Insect DNA Kit (OMEGA Bio-Tek, D0926-01, Guangzhou, China) according to the manufacturer's standard protocol. PCR was performed to amplify a 709 bp fragment of the 5' *cox*1 region of mtDNA using the DNA primer pairs LCO1490 (5' GGTCAACAAATCATAAAGATATTGG-3') and HCO2198 (5'-TAAACTTCAGGGTGACCAAAAAATCA-3') [2, 3]. PCR amplification was performed in a 50μl reaction volume with 1μl 5×TransStart® FastPfu Fly DNA Polymerase (TransGen Biotech, Beijing, China), 5μl 2.5 mmol/l tNTPs, 1 μl each of the forward and reverse primers at 10 μmol/l, 2 μl of template DNA and sufficient nuclease-free water to make 50 μl. PCR conditions were as follows: an initial denaturation at 98 °C for 2 min followed by five cycles of 98 °C for 10 s (denaturation), 50 °C for 20 s (annealing), and 72 °C for 15 s (extension); 40 cycles of 98 °C for 10 s (denaturation), 50 °C for 20 s (annealing), and 72 °C for 15 s (extension); and a final extension at 72 °C for 5 min. The amplified fragments were run on a 1.5% agarose gel to check integrity, stained with ethidium bromide and analyzed under UV light. PCR products were sequenced with PCR primers in both directions using the ABI 3730XL automatic sequencer (Ige Biotechnology, Guangzhou, China). Phylogenetic trees were constructed based on the aligned nucleotide sequences using the neighbor-joining (NJ) method *via* the maximum composite likelihood substitution model in MEGA 7.0.26 [4].

Results

In this field investigation in Guangzhou, three species (*Ae. albopictus, Cx.*

quinquefasciatus and *Ar. subalbatus*) of mosquitoes have identified from morphology. We further confirmed the results from PCR (Text S2a) and sequence analyses (Text S2b) of *cox*1 gene. Of the 38 individuals of *Ae. albopictus*, 38 sequences (100%) were identical similarity with *Ae. Albopictus* (Text S2b). Of the 38 individuals of *Cx. quinquefasciatus*, 38 sequences (100%) were identical similarity with *Cx. quinquefasciatus* (Text S2b). Of the 2 individuals of *Ar. subalbatus*, 2 sequences (100%) were identical similarity with *Ar. subalbatus* (Text S2b).

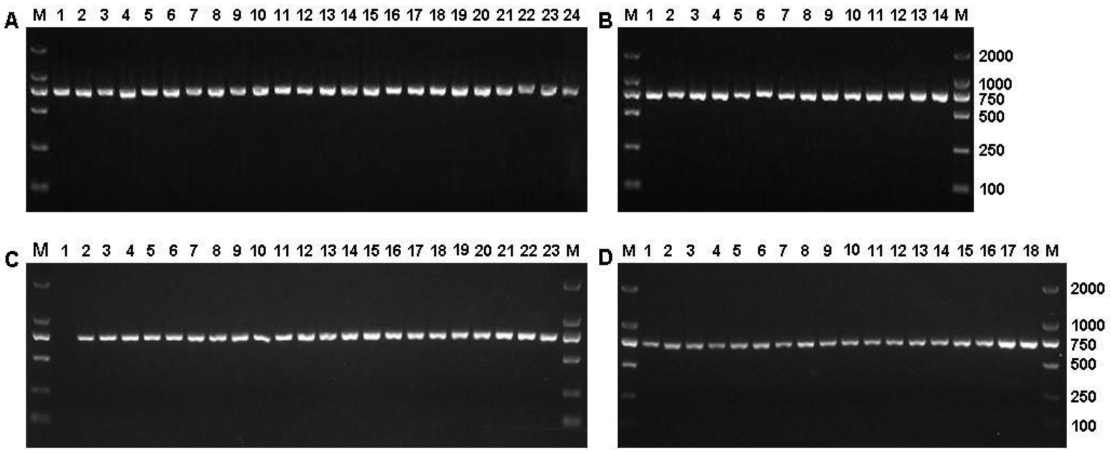

Text S2a. PCR identification using *cox*1 gene of *Ae. albopictus*, *Cx. quinquefasciatus* and *Ar. subalbatus*. All PCR products except lane1 from C (blank control) are directly amplified from total DNA extraction. A, B PCR fragments of *cox*1 gene in 38 *Ae. albopictus*; C Agarose gel of PCR products from *cox*1 gene of 23 *Cx. quinquefasciatus*; D PCR fragments of *cox*1 gene in 15 *Cx. quinquefasciatus*. Except lane 17 and 18 are the products of *cox*1 gene in 2 *Ar. subalbatus*.

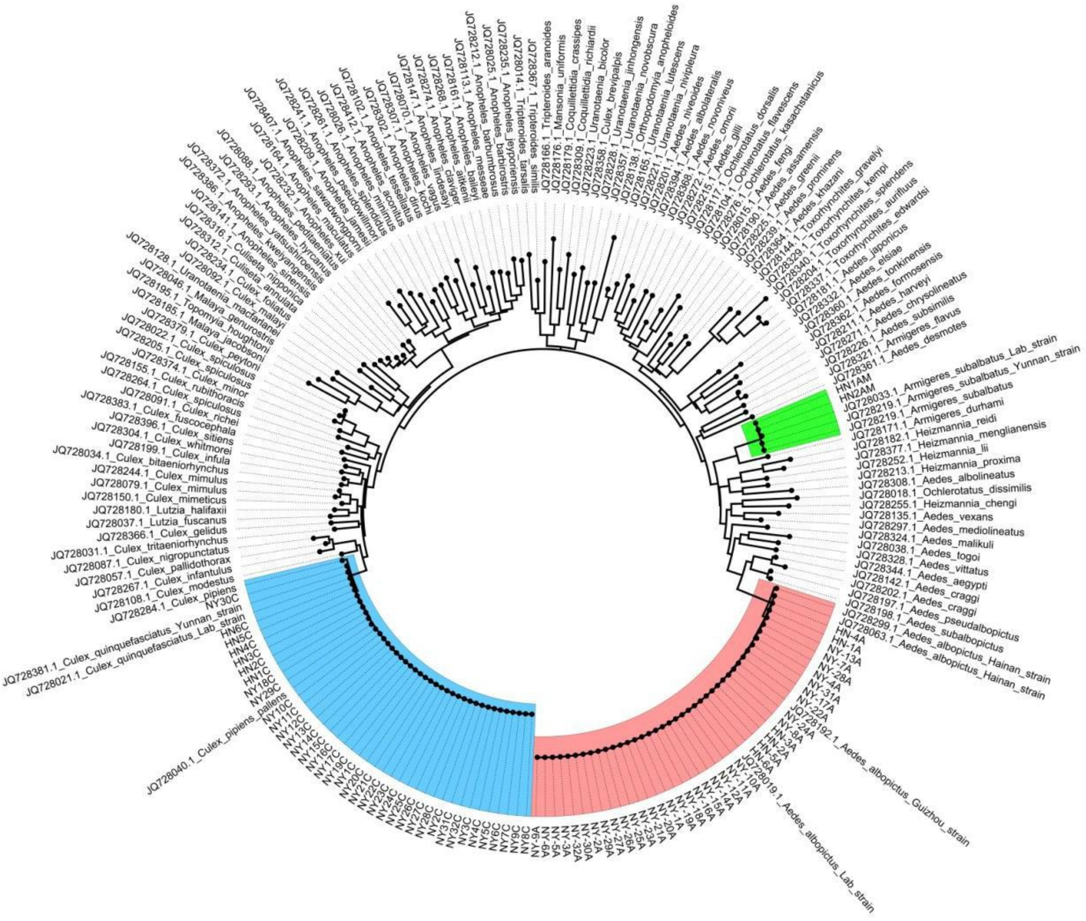

Text S2b. NJ phylogenetic tree based on *cox*1 gene sequences of mosquitoes prevalent in China and the collected adult mosquitoes. The sequences named as HY1A-32A, HN1A-6A are *cox*1 gene of mosquitoes identified as *Ae. albopictus* by morphology. The sequences named as HY1C-32C, HN1C-6C are *cox*1 gene of mosquitoes identified as *Cx. quinquefasciatus* by morphology. The sequences named as HN1AM and HN2AM are *cox*1 gene of mosquitoes identified as *Ar. subalbatus* by morphology. Branches on the tree of *Ae. albopictus*, *Cx. quinquefasciatus* and *Ar. subalbatus* were marked with pink, blue and green shadow, respectively.

References

1. Lu BL, Li BS, Ji SH, Chen HB, Meng QH, Su L, et al. Fauna Sinica, Insecta vol. 8, Diptera: Culicidae 1. Beijing, China: Science Press; 1997.
2. Folmer O, Black M, Hoeh W, Lutz R, Vrijenhoek R. DNA primers for amplification of mitochondrial cytochrome c oxidase subunit I from diverse metazoan invertebrates. Mol Mar Biol Biotechnol. 1994; 3: 294–9.
3. Wang G, Li CX, Guo XX, Xing D, Dong YD, Wang ZM, et al. Identifying the main mosquito species in China based on DNA barcoding. PLoS One. 2012; 7: e47051.
4. Kumar S, Stecher G, Tamura K. MEGA7: Molecular Evolutionary Genetics Analysis Version 7.0 for Bigger Datasets. Mol Biol Evol. 2016; 33: 1870–4.
