# Supplementary material for: A field-based modeling study on ecological characterization of hourly host-seeking behavior and its associated climatic variables in Aedes albopictus
Source: Parasit Vectors. 2019 Oct 14;12:474. doi: 10.1186/s13071-019-3715-1 (PMC6791010; doi:10.1186/s13071-019-3715-1)
Supplement: Supplementary file 3 — Additional file 3: Table S1. The fixed values of other variables. [file 13071_2019_3715_MOESM3_ESM.pdf]

Table S1. The fixed values of other variables.

Variable	Value
Time points within a day	18:00
Month	June
Temperature (°C)	25.2
Relative humidity (%)	82
Whether the time point was in the daytime or at nighttime	In the daytime
Wind speed (m/s)	0.4
