# Supplementary material for: A field-based modeling study on ecological characterization of hourly host-seeking behavior and its associated climatic variables in Aedes albopictus
Source: Parasit Vectors. 2019 Oct 14;12:474. doi: 10.1186/s13071-019-3715-1 (PMC6791010; doi:10.1186/s13071-019-3715-1)
Supplement: Supplementary file 4 — Additional file 4: Table S2. Adult mosquitoes collected by multi-month investigations from November 2016 to November 2017. [file 13071_2019_3715_MOESM4_ESM.pdf]

Table S2. Adult mosquitoes collected by multi-month field investigations from November 2016 to November 2017

	Female *Ae. albopictus*		Male *Ae. albopictus*		Female *Cx. quinquefasciatus*		Male *Cx. quinquefasciatus*		
Month and year	Daytime	Nighttime	Daytime	Nighttime	Daytime	Nighttime	Daytime	Nighttime	Total
2016-Nov	222	17	67	0	3	40	2	24	375
2016-Dec	10	1	1	0	2	26	2	6	48
2017-Jan	14	3	2	0	44	199	26	37	325
2017-Feb	3	0	0	0	3	43	21	13	83
2017-Mar	2	1	0	0	10	13	1	0	27
2017-Apr	45	10	33	4	5	19	1	0	123
2017-May	180	61	101	11	2	12	1	0	368
2017-Jun	220	92	149	26	2	47	1	0	537
2017-Jul	118	33	137	34	0	39	1	3	365
2017-Aug	28	31	33	15	10	26	0	3	146
2017-Sep	44	24	43	5	1	22	0	4	143
2017-Oct	60	41	49	24	5	17	0	2	198
2017-Nov	12	9	3	1	1	72	2	14	114
Total	958	329	618	120	88	575	58	106	2852
