# Supplementary material for: A field-based modeling study on ecological characterization of hourly host-seeking behavior and its associated climatic variables in Aedes albopictus
Source: Parasit Vectors. 2019 Oct 14;12:474. doi: 10.1186/s13071-019-3715-1 (PMC6791010; doi:10.1186/s13071-019-3715-1)
Supplement: Supplementary file 5 — Additional file 5: Table S3. Adult mosquitoes collected by multi-site investigations during June-July 2018. [file 13071_2019_3715_MOESM5_ESM.pdf]

Table S3. Adult mosquitoes collected by multi-site field investigations during June-July 2018

	Female *Ae. albopictus*		Male *Ae. albopictus*		Female *Cx. quinquefasciatus*		Male *Cx. quinquefasciatus*		Female *Ar. subalbatus*		
Area	Daytime	Nighttime	Daytime	Nighttime	Daytime	Nighttime	Daytime	Nighttime	Daytime	Nighttime	Total
Main Site	487	108	550	142	16	70	6	10	0	0	1389
Site A	543	233	268	130	32	96	6	14	0	0	1322
Site B	160	144	214	38	22	38	4	2	18	6	646
Site C	152	115	157	103	2	40	6	2	4	0	581
Total	1342	600	1189	413	72	244	22	28	22	6	3938
