# Supplementary material for: A field-based modeling study on ecological characterization of hourly host-seeking behavior and its associated climatic variables in Aedes albopictus
Source: Parasit Vectors. 2019 Oct 14;12:474. doi: 10.1186/s13071-019-3715-1 (PMC6791010; doi:10.1186/s13071-019-3715-1)
Supplement: Supplementary file 6 — Additional file 6: Table S4. The predicted values for the multi-month and multi-site mosquito densities of Ae. albopictus during both the day and night. [file 13071_2019_3715_MOESM6_ESM.pdf]

Table S4a. The predicted value for the multi-month mosquito density of *Ae. albopictus* during both the day and night

	Female *Ae.albopictus*		Male *Ae. albopictus*	
	Density	95% CrI	Density	95% CrI
Daytime	24.4	(11.9, 50.0)	6.5	(2.3, 19.6)
Nighttime	5.5	(2.4, 12.6)	0.7	(0.2, 2.6)

Abbreviations: 95% CrI, 95% credible interval; Density, mosquito number per person per hour.

Table S4b. The predicted value for the multi-site mosquito density of *Ae. albopictus* during both the day and night

	Female *Ae.albopictus*		Male *Ae. albopictus*	
	Density	95% CrI	Density	95% CrI
Daytime	34.0	(7.7, 167.2)	66.0	(12.9, 385.9)
Nighttime	16.5	(3.6, 88.1)	16.1	(2.9, 101.2)

Abbreviations: 95% CrI, 95% credible interval; Density, mosquito number per person per hour.
