# Supplementary figures and images for: A field-based modeling study on ecological characterization of hourly host-seeking behavior and its associated climatic variables in Aedes albopictus
Source: Parasit Vectors. 2019 Oct 14;12:474. doi: 10.1186/s13071-019-3715-1 (PMC6791010; doi:10.1186/s13071-019-3715-1)

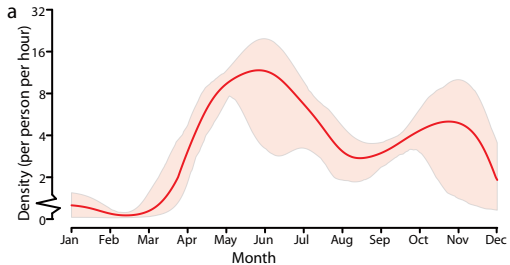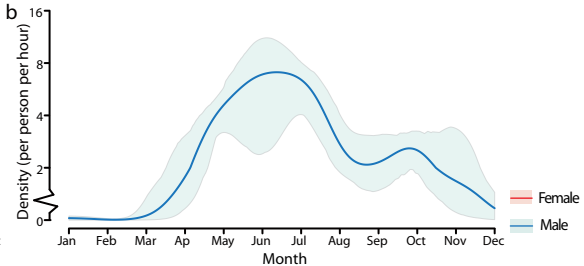

Supplement: Supplementary file 7 — Additional file 7: Figure S1. Seasonal variations of hourly host-seeking activities found in Ae. albopictus during 2016–2017. a, b Seasonal variations of hourly host-seeking activities found in female (a) and male (b) Ae. albopictus. The smoothed hourly densities of female and male Ae. albopictus are displayed in solid red and blue lines, respectively. The shaded areas represent 95% credible intervals of the smoothed hourly densities. [file 13071_2019_3715_MOESM7_ESM.pdf]
