# Supplementary material for: A field-based modeling study on ecological characterization of hourly host-seeking behavior and its associated climatic variables in Aedes albopictus
Source: Parasit Vectors. 2019 Oct 14;12:474. doi: 10.1186/s13071-019-3715-1 (PMC6791010; doi:10.1186/s13071-019-3715-1)
Supplement: Supplementary file 8 — Additional file 8: Table S5. Values of the Watanabe-Akaike information criterion (WAIC) for models used for the assessment of potential influential factors of Ae. albopictus for the period from November 2016 to November 2017. [file 13071_2019_3715_MOESM8_ESM.pdf]

Table S5a. Values of the Watanabe-Akaike information criterion (WAIC) for models used for the assessment of potential influential factors (without illuminance) of female *Ae. albopictus* for the period from November 2016 to November 2017.

Included terms																							
Intercept	*Month*	*ns*(TPWAD)		*ns*(*Temp*)						*ns*(*RH*)						*d_or_n*	*ns*(*Wind*)						WAIC
		df=4	*df*=6	*df*=1	*df*=2	*df*=3	*df*=4	*df*=5	*df*=6	*df*=1	*df*=2	*df*=3	*df*=4	*df*=5	*df*=6		*df*=1	*df*=2	*df*=3	*df*=4	*df*=5	*df*=6	
✓	✓	✓																					2476.75
✓	✓		✓																				2468.72
✓	✓	✓		✓																			2479.30
✓	✓	✓			✓																		2381.97
✓	✓	✓				✓																	2385.42
✓	✓	✓					✓																2386.50
✓	✓	✓						✓															2388.87
✓	✓	✓							✓														2391.65
✓	✓	✓								✓													2451.59
✓	✓	✓									✓												2454.13
✓	✓	✓										✓											2453.98
✓	✓	✓											✓										2455.17
✓	✓	✓												✓									2439.66
✓	✓	✓													✓								2433.45
✓	✓	✓														✓							2454.28
✓	✓	✓															✓						2468.04
✓	✓	✓																✓					2467.21
✓	✓	✓																	✓				2467.91
✓	✓	✓																		✓			2455.54
✓	✓	✓																			✓		2454.94
✓	✓	✓																				✓	2455.57
✓	✓	✓			✓					✓													2378.66
✓	✓	✓			✓						✓												2380.82
✓	✓	✓			✓							✓											2382.42
✓	✓	✓			✓								✓										2383.99
✓	✓	✓			✓									✓									2376.05
✓	✓	✓			✓										✓								2369.54
✓	✓	✓			✓					✓						✓							2350.90
✓	✓	✓			✓					✓						✓	✓						2352.17
✓	✓	✓			✓					✓						✓		✓					2351.50
✓	✓	✓			✓					✓						✓			✓				2351.68
**✓**	**✓**	**✓**			**✓**					**✓**						**✓**				**✓**			**2334.14**
✓	✓	✓			✓					✓						✓					✓		2333.66
✓	✓	✓			✓					✓						✓						✓	2334.64

Month represents a categorical variable indicating month. *ns*(.) means natural cubic spline function of a variable. TPWAD represents the time point within a day. *Temp*, *RH*, and *Wind* represent temperature, relative humidity, and wind speed, respectively. *df* means degree of freedom. *d_or_n* represents whether the time point is in the daytime or at nighttime.
Note. The final model was in bold.

Table S5b. Values of the Watanabe-Akaike information criterion (WAIC) for models used for the assessment of potential influential factors (with illuminance) of female *Ae. albopictus* for the period from between November 2016 to November 2017.

Included terms														WAIC
Intercept	*Month*	*ns*(TPWAD, *df*=4)	*ns*(log(*Illum*))					*ns*(*Wind*)						
			df=2	*df*=3	*df*=4	*df*=5	*df*=6	*df*=1	*df*=2	*df*=3	*df*=4	*df*=5	*df*=6	
✓	✓	✓	✓											2456.82
✓	✓	✓		✓										2420.02
✓	✓	✓			✓									2410.93
✓	✓	✓				✓								2414.91
✓	✓	✓					✓							2412.93
✓	✓	✓		✓				✓						2410.47
✓	✓	✓		✓					✓					2409.33
✓	✓	✓		✓						✓				2409.47
**✓**	**✓**	**✓**		**✓**							**✓**			**2397.81**
✓	✓	✓		✓								✓		2398.36
✓	✓	✓		✓									✓	2400.08

Month represents a categorical variable indicating month. *ns*(.) means natural cubic spline function of a variable. TPWAD represents the time point within a day. *df* means degree of freedom. log(*Illum*+0.001) means the log-transformation of (*Illum* + 0.001).
Note. The final model was in bold.

Table S5c. Values of the Watanabe-Akaike information criterion (WAIC) for models used for the assessment of potential influential factors (without illuminance) of male *Ae. albopictus* for the period from November 2016 to November 2017.

Included terms																							WAIC
Intercept	*Month*	*ns*(TPWAD)		*ns*(*Temp*)						*ns*(*RH*)						*d_or_n*	*ns*(*Wind*)						
		df=4	*df*=6	*df*=1	*df*=2	*df*=3	*df*=4	*df*=5	*df*=6	*df*=1	*df*=2	*df*=3	*df*=4	*df*=5	*df*=6		*df*=1	*df*=2	*df*=3	*df*=4	*df*=5	*df*=6	
✓	✓	✓																					1641.24
✓	✓		✓																				1615.56
✓	✓		✓	✓																			1618.93
✓	✓		✓		✓																		1542.41
✓	✓		✓			✓																	1542.95
✓	✓		✓				✓																1544.94
✓	✓		✓					✓															1546.99
✓	✓		✓						✓														1546.34
✓	✓		✓							✓													1599.56
✓	✓		✓								✓												1594.54
✓	✓		✓									✓											1596.11
✓	✓		✓										✓										1597.99
✓	✓		✓											✓									1594.08
✓	✓		✓												✓								1596.56
✓	✓		✓													✓							1601.58
✓	✓		✓														✓						1611.65
✓	✓		✓															✓					1613.15
✓	✓		✓																✓				1612.90
✓	✓		✓																	✓			1612.80
✓	✓		✓																		✓		1614.62
✓	✓		✓																			✓	1614.54
✓	✓		✓		✓					✓													1538.15
✓	✓		✓		✓						✓												1539.45
✓	✓		✓		✓							✓											1541.19
✓	✓		✓		✓								✓										1544.30
✓	✓		✓		✓									✓									1542.46
✓	✓		✓		✓										✓								1543.74
**✓**	**✓**		**✓**		**✓**					**✓**						**✓**							**1520.58**
✓	✓		✓		✓					✓						✓	✓						1522.07
✓	✓		✓		✓					✓						✓		✓					1522.51
✓	✓		✓		✓					✓						✓			✓				1521.85
✓	✓		✓		✓					✓						✓				✓			1522.41
✓	✓		✓		✓					✓						✓					✓		1524.38
✓	✓		✓		✓					✓						✓						✓	1524.74

Month represents a categorical variable indicating month. *ns*(.) means natural cubic spline function of a variable. TPWAD represents the time point within a day. *Temp*, *RH*, and *Wind* represent temperature, relative humidity, and wind speed, respectively. *df* means degree of freedom. *d_or_n* represents whether the time point is in the daytime or at nighttime.
Note. The final model was in bold.

Table S5d. Values of the Watanabe-Akaike information criterion (WAIC) for models used for the assessment of potential influential factors (with illuminance) of male *Ae. Albopictus* for the period from November 2016 to November 2017.

Included terms														WAIC
Intercept	*Month*	*ns*(TPWAD, *df*=6)	*ns*(log(*Illum*))					*ns*(*Wind*)						
			df=2	*df*=3	*df*=4	*df*=5	*df*=6	*df*=1	*df*=2	*df*=3	*df*=4	*df*=5	*df*=6	
✓	✓	✓	✓											1594.09
**✓**	**✓**	**✓**		**✓**										**1574.23**
✓	✓	✓			✓									1573.75
✓	✓	✓				✓								1571.36
✓	✓	✓					✓							1575.41
✓	✓	✓		✓				✓						1573.34
✓	✓	✓		✓					✓					1575.10
✓	✓	✓		✓						✓				1574.98
✓	✓	✓		✓							✓			1575.94
✓	✓	✓		✓								✓		1577.64
✓	✓	✓		✓									✓	1577.76

Month represents a categorical variable indicating month. *ns*(.) means natural cubic spline function of a variable. TPWAD represents the time point within a day. *df* means degree of freedom. log(*Illum*+0.001) means the log-transformation of (*Illum* + 0.001).
Note. The final model was in bold.
