# Supplementary material for: A field-based modeling study on ecological characterization of hourly host-seeking behavior and its associated climatic variables in Aedes albopictus
Source: Parasit Vectors. 2019 Oct 14;12:474. doi: 10.1186/s13071-019-3715-1 (PMC6791010; doi:10.1186/s13071-019-3715-1)
Supplement: Supplementary file 9 — Additional file 9: Table S6. R2 values for the models with time points within a day and month and different variables. [file 13071_2019_3715_MOESM9_ESM.pdf]

Table S6. R^2 values for the models with time points within a day and month and different variables.

Variables	R^2 (%)			
	Female *Ae. albopictus*	(95% CrI)	Male *Ae. albopictus*	(95% CrI)
Temp	34.6	(23.3, 46.6)	51.7	(36.1, 58.8)
RH	37.8	(24.2, 51.0)	51.2	(34.2, 56.2)
d_or_n	37.1	(23.9, 50.5)	45.3	(26.8, 59.1)
Wind	36.8	(24.3, 48.3)	-	-
log(*Illum*+0.001)	45.6	(32.2, 54.3)	51.5	(35.4, 58.5)
Temp + *RH* + *d_or_n*	-	-	58.5	(44.2, 64.2)
Temp + *RH* + *d_or_n* + *Wind*	45.6	(34.7, 54.5)	-	-
log(*Illum*+0.001) + *Wind*	46.3	(35.0, 54.2)	-	-

Abbreviation: 95% CrI, 95% credible interval.

Temp means temperature. *RH* means relative humidity. *d_or_n* means whether the time point is in the daytime or at nighttime. *Wind* means wind speed. log(*Illum*+0.001) means the log-transformation of (*Illum* + 0.001).

- means the R^2 was not calculated for the model which was not fitted.
