# Supplementary material for: A field-based modeling study on ecological characterization of hourly host-seeking behavior and its associated climatic variables in Aedes albopictus
Source: Parasit Vectors. 2019 Oct 14;12:474. doi: 10.1186/s13071-019-3715-1 (PMC6791010; doi:10.1186/s13071-019-3715-1)
Supplement: Supplementary file 10 — Additional file 10: Table S7. Values of the Watanabe-Akaike information criterion (WAIC) for models used for the assessment of potential influential factors of Ae. albopictus for the period June-July 2018. [file 13071_2019_3715_MOESM10_ESM.pdf]

Table S7a. Values of the Watanabe-Akaike information criterion (WAIC) for models used for the assessment of potential influential factors (without illuminance) of female *Ae. albopictus* for the period June-July 2018.

Included terms																							WAIC
Intercept	*Month*	*ns*(TPWAD)		*ns*(*Temp*)						*ns*(*RH*)						*d_or_n*	*ns*(*Wind*)						
		df=4	*df*=6	*df*=1	*df*=2	*df*=3	*df*=4	*df*=5	*df*=6	*df*=1	*df*=2	*df*=3	*df*=4	*df*=5	*df*=6		*df*=1	*df*=2	*df*=3	*df*=4	*df*=5	*df*=6	
✓	✓	✓																					2010.73
✓	✓		✓																				1984.78
✓	✓		✓	✓																			1953.59
✓	✓		✓		✓																		1955.09
✓	✓		✓			✓																	1957.72
✓	✓		✓				✓																1958.72
✓	✓		✓					✓															1960.91
✓	✓		✓						✓														1962.13
✓	✓		✓							✓													1959.70
✓	✓		✓								✓												1959.48
✓	✓		✓									✓											1962.35
✓	✓		✓										✓										1960.30
✓	✓		✓											✓									1963.61
✓	✓		✓												✓								1955.55
✓	✓		✓													✓							1982.01
✓	✓		✓														✓						1987.28
✓	✓		✓															✓					1989.25
✓	✓		✓																✓				1988.25
✓	✓		✓																	✓			1989.30
✓	✓		✓																		✓		1988.94
✓	✓		✓																			✓	1986.40
✓	✓		✓	✓						✓													1955.24
✓	✓		✓	✓							✓												1958.56
✓	✓		✓	✓								✓											1961.59
✓	✓		✓	✓									✓										1953.40
✓	✓		✓	✓										✓									1957.83
✓	✓		✓	✓											✓								1952.55
**✓**	**✓**		**✓**	**✓**												**✓**							**1951.44**
✓	✓		✓	✓												✓	✓						1954.07
✓	✓		✓	✓												✓		✓					1951.90
✓	✓		✓	✓												✓			✓				1950.92
✓	✓		✓	✓												✓				✓			1956.94
✓	✓		✓	✓												✓					✓		1952.64
✓	✓		✓	✓												✓						✓	1949.34

Month represents a categorical variable indicating month. *ns*(.) means natural cubic spline function of a variable. TPWAD represents the time point within a day. *Temp*, *RH*, and *Wind* represent temperature, relative humidity, and wind speed, respectively. *df* means degree of freedom. *d_or_n* represents whether the time point is in the daytime or at nighttime.

Note. The final model was in bold.

Table S7b. Values of the Watanabe-Akaike information criterion (WAIC) for models used for the assessment of potential influential factors (without illuminance) of male *Ae. albopictus* for the period June-July 2018.

Included terms																							WAIC
		ns(TPWAD)		*ns*(*Temp*)						*ns*(*RH*)						*d_or_n*	*ns*(*Wind*)						
Intercept	*Month*	*df*=4	*df*=6	*df*=1	*df*=2	*df*=3	*df*=4	*df*=5	*df*=6	*df*=1	*df*=2	*df*=3	*df*=4	*df*=5	*df*=6		*df*=1	*df*=2	*df*=3	*df*=4	*df*=5	*df*=6	
✓	✓	✓																					1801.25
✓	✓		✓																				1789.52
✓	✓	✓		✓																			1746.67
✓	✓	✓			✓																		1737.94
✓	✓	✓				✓																	1739.64
✓	✓	✓					✓																1740.83
✓	✓	✓						✓															1742.58
✓	✓	✓							✓														1743.68
✓	✓	✓								✓													1766.22
✓	✓	✓									✓												1750.87
✓	✓	✓										✓											1748.84
✓	✓	✓											✓										1748.20
✓	✓	✓												✓									1750.55
✓	✓	✓													✓								1744.60
✓	✓	✓														✓							1782.22
✓	✓	✓															✓						1802.69
✓	✓	✓																✓					1802.02
✓	✓	✓																	✓				1801.02
✓	✓	✓																		✓			1804.84
✓	✓	✓																			✓		1804.05
✓	✓	✓																				✓	1803.65
✓	✓	✓		✓						✓													1748.11
✓	✓	✓		✓							✓												1742.74
✓	✓	✓		✓								✓											1738.94
✓	✓	✓		✓									✓										1733.37
✓	✓	✓		✓										✓									1738.36
✓	✓	✓		✓											✓								1730.78
**✓**	**✓**	**✓**		**✓**												**✓**							**1727.81**
✓	✓	✓		✓												✓	✓						1729.69
✓	✓	✓		✓												✓		✓					1730.01
✓	✓	✓		✓												✓			✓				1728.60
✓	✓	✓		✓												✓				✓			1731.70
✓	✓	✓		✓												✓					✓		1730.45
✓	✓	✓		✓												✓						✓	1731.31

Month represents a categorical variable indicating month. *ns*(.) means natural cubic spline function of a variable. TPWAD represents the time point within a day. *Temp*, *RH*, and *Wind* represent temperature, relative humidity, and wind speed, respectively. *df* means degree of freedom. *d_or_n* represents whether the time point is in the daytime or at nighttime.

Note. The final model was in bold.

Table S7c. Values of the Watanabe-Akaike information criterion (WAIC) for models used for the assessment of potential influential factors (with illuminance) of female *Ae. albopictus* for the period June-July 2018.

Included terms														
Intercept	*Month*	*ns*(TPWAD, *df*=6)	*ns*(log(*Illum*))					*ns*(*Wind*)						WAIC
			df=2	*df*=3	*df*=4	*df*=5	*df*=6	*df*=1	*df*=2	*df*=3	*df*=4	*df*=5	*df*=6	
✓	✓	✓	✓											**1978.57**
✓	✓	✓		✓										1975.06
✓	✓	✓			✓									1976.67
✓	✓	✓				✓								1978.12
✓	✓	✓					✓							1979.31
✓	✓	✓	✓					✓						1980.82
✓	✓	✓	✓						✓					1983.16
✓	✓	✓	✓							✓				1980.36
✓	✓	✓	✓								✓			1984.85
✓	✓	✓	✓									✓		1980.83
✓	✓	✓	✓										✓	1981.30

Month represents a categorical variable indicating month. *ns*(.) means natural cubic spline function of a variable. TPWAD represents the time point within a day. *df* means degree of freedom. log(*Illum*+0.001) means the log-transformation of (*Illum* + 0.001).
Note. The final model was in bold.

Table S7d. Values of the Watanabe-Akaike information criterion (WAIC) for models used for the assessment of potential influential factors (with illuminance) of male *Ae. albopictus* for the period June-July 2018.

Included terms														WAIC
Intercept	*Month*	*ns*(TPWAD, *df*=4)	*ns*(log(*Illum*))					*ns*(*Wind*)						
			df=2	*df*=3	*df*=4	*df*=5	*df*=6	*df*=1	*df*=2	*df*=3	*df*=4	*df*=5	*df*=6	
✓	✓	✓	✓											1793.27
✓	✓	✓		✓										**1756.29**
✓	✓	✓			✓									1758.95
✓	✓	✓				✓								1760.99
✓	✓	✓					✓							1759.68
✓	✓	✓		✓				✓						1758.24
✓	✓	✓		✓					✓					1759.01
✓	✓	✓		✓						✓				1758.75
✓	✓	✓		✓							✓			1761.31
✓	✓	✓		✓								✓		1763.45
✓	✓	✓		✓									✓	1760.15

Month represents a categorical variable indicating month. *ns*(.) means natural cubic spline function of a variable. TPWAD represents the time point within a day. *df* means degree of freedom. log(*Illum*+0.001) means the log-transformation of (*Illum* + 0.001).
Note. The final model was in bold.
