# Supplementary material for: A field-based modeling study on ecological characterization of hourly host-seeking behavior and its associated climatic variables in Aedes albopictus
Source: Parasit Vectors. 2019 Oct 14;12:474. doi: 10.1186/s13071-019-3715-1 (PMC6791010; doi:10.1186/s13071-019-3715-1)
Supplement: Supplementary file 11 — Additional file 11: Figure S2. The histograms and trace plots of the parameter values. [file 13071_2019_3715_MOESM11_ESM.pdf]

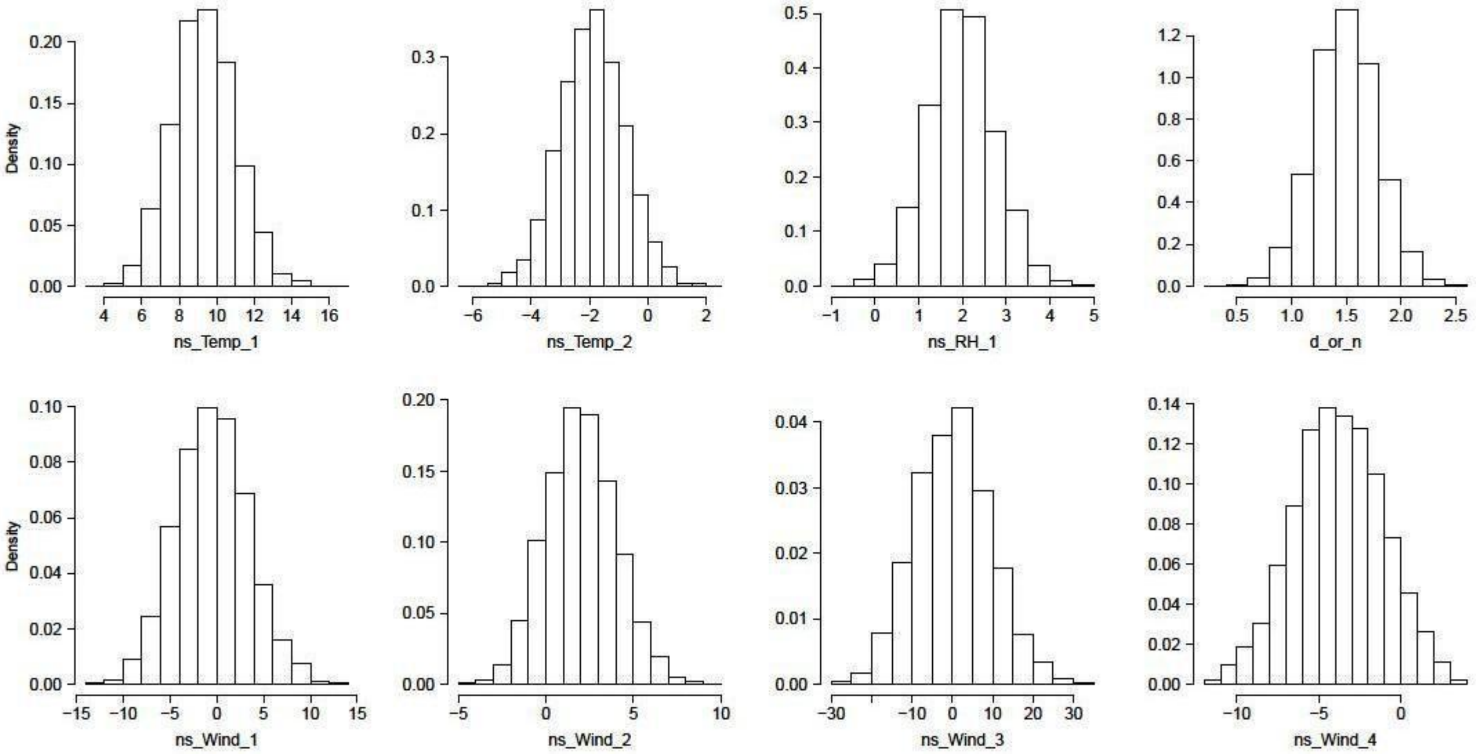

Figure S2a. Histograms of the parameter values of temperature, relative humidity, whether the time point was in the daytime or at night, and wind speed (excluding those from warm-up iterations) in the model fitted to female mosquito densities during November 2016 to November 2017. ns_Temp_k (k=1, 2) indicates the term of a natural cubic spline of temperature; ns_RH_1 indicates the term of a natural cubic spline of relative humidity; d_or_n indicates whether the time point was in the daytime or at night; ns_Wind_k (k=1, 2, 3, 4) indicates the term of a natural cubic spline of wind speed.

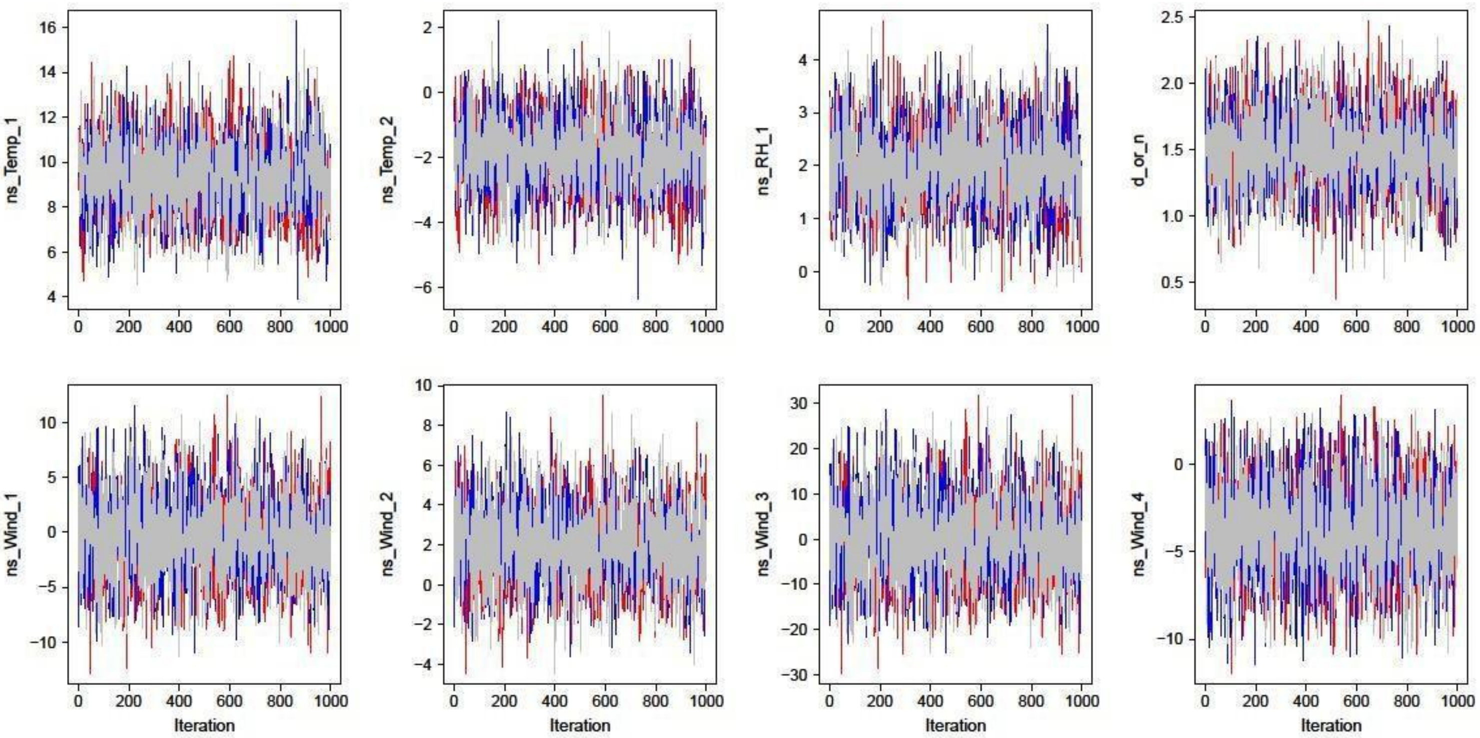

Figure S2b. Trace plots of the parameter values of temperature, relative humidity, whether the time point was in the daytime or at night, and wind speed (excluding those from warm-up iterations) in the model fitted to female mosquito densities during November 2016 to November 2017 from chain 1 (red lines), chain 2 (blue lines), and chain 3 (grey lines). ns_Temp_k (k=1, 2) indicates the term of a natural cubic spline of temperature; ns_RH_1 indicates the term of a natural cubic spline of relative humidity; d_or_n indicates whether the time point was in the daytime or at night; ns_Wind_k (k=1, 2, 3, 4) indicates the term of a natural cubic spline of wind speed.

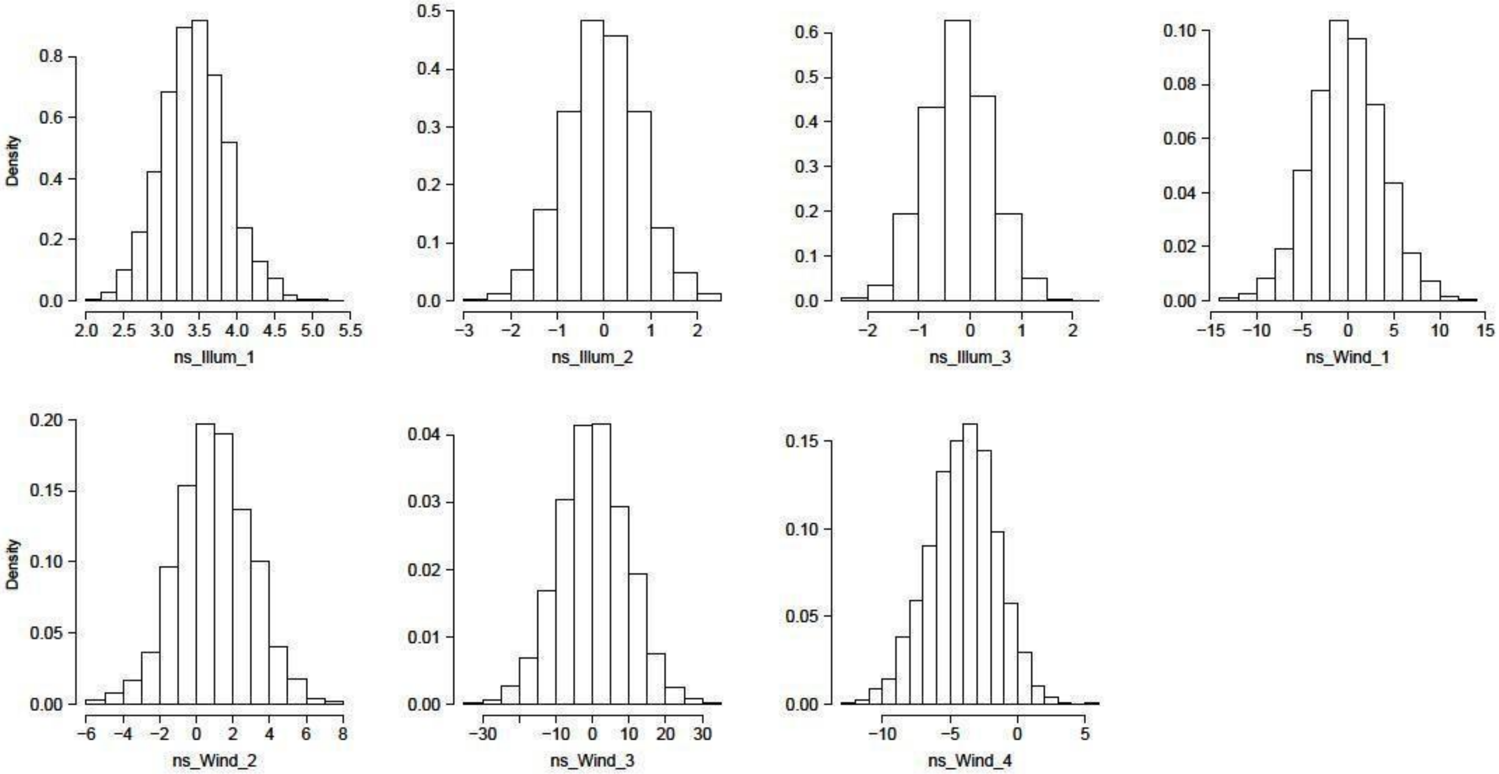

Figure S2c. Histograms of the parameter values of illuminance and wind speed (excluding those from warm-up iterations) in the model fitted to female mosquito densities during November 2016 to November 2017. ns_Illum_k (k=1, 2, 3) indicates the term of a natural cubic spline of illuminance; ns_Wind_k (k=1, 2, 3, 4) indicates the term of a natural cubic spline of wind speed.

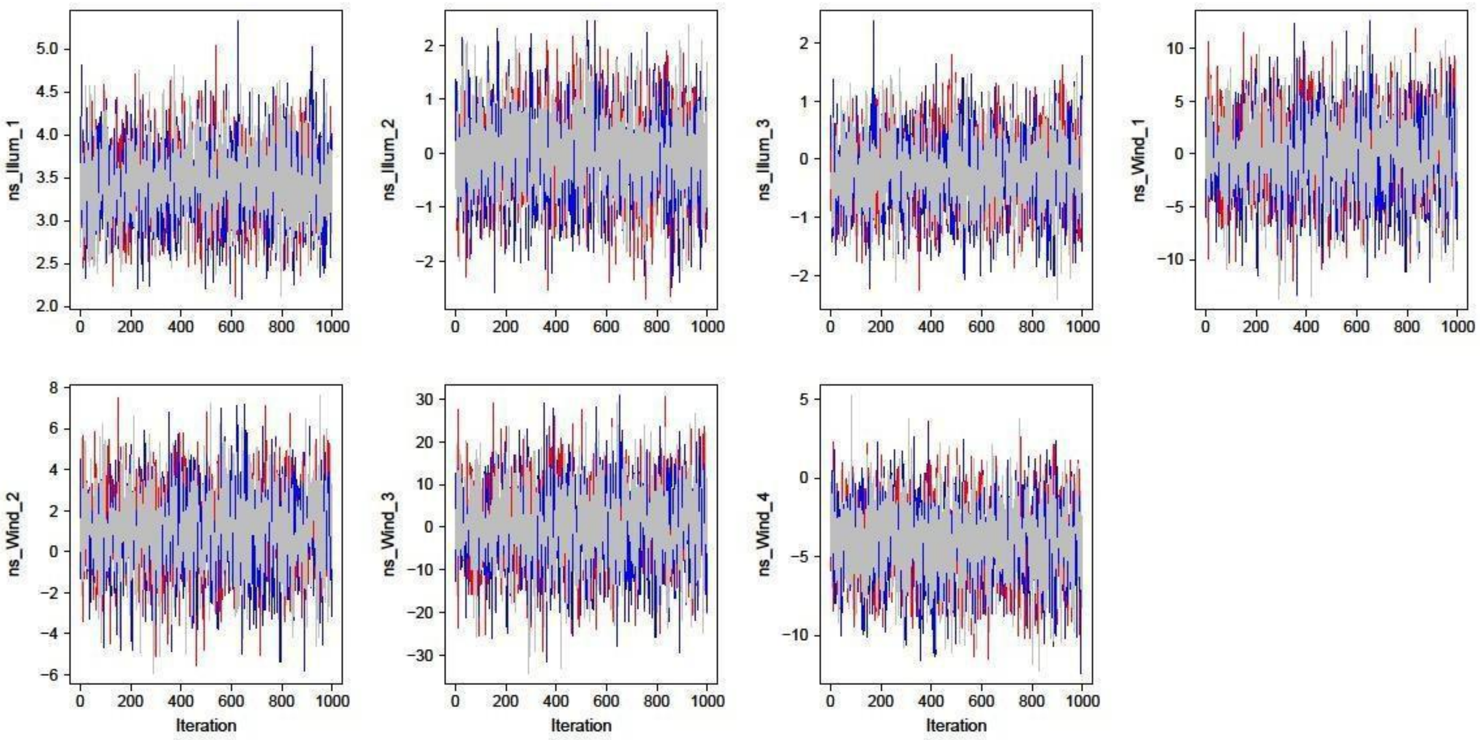

Figure S2d. Trace plots of the parameter values of illuminance and wind speed (excluding those from warm-up iterations) in the model fitted to female mosquito densities during November 2016 to November 2017 from chain 1 (red lines), chain 2 (blue lines), and chain 3 (grey lines). ns_Illum_k (k=1, 2, 3) indicates the term of a natural cubic spline of illuminance; ns_Wind_k (k=1, 2, 3, 4) indicates the term of a natural cubic spline of wind speed.

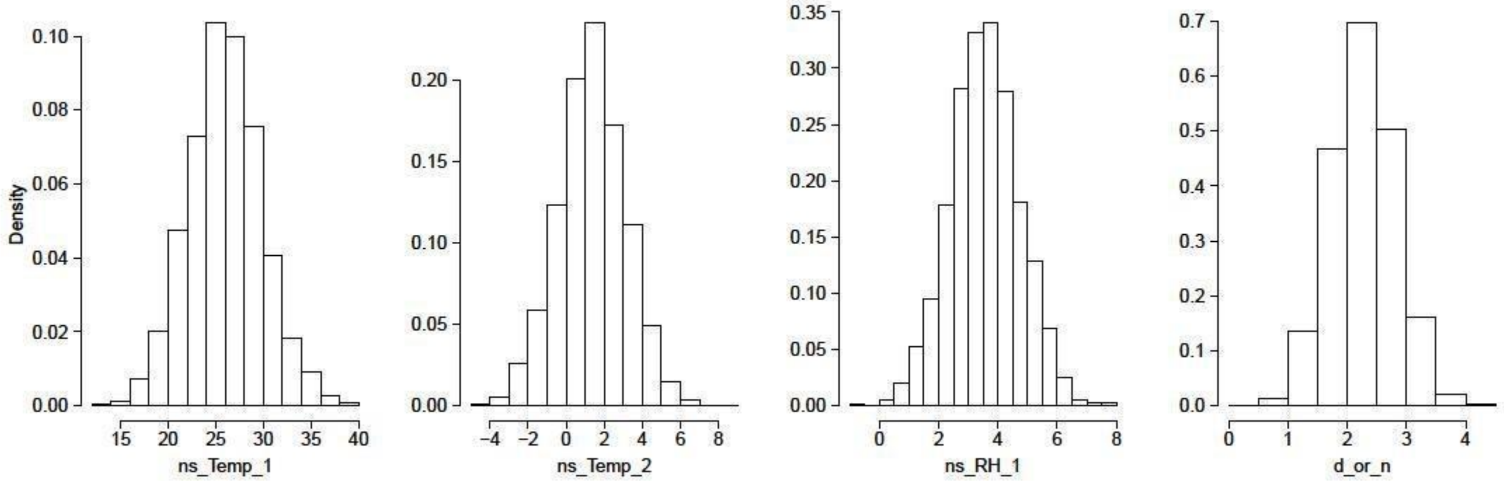

Figure S2e. Histograms of the parameter values of temperature, relative humidity, and whether the time point was in the daytime or at night (excluding those from warm-up iterations) in the model fitted to male mosquito densities during November 2016 to November 2017. ns_Temp_k (k=1, 2) indicates the term of a natural cubic spline of temperature; ns_RH_1 indicates the term of a natural cubic spline of relative humidity; d_or_n indicates whether the time point was in the daytime or at night.

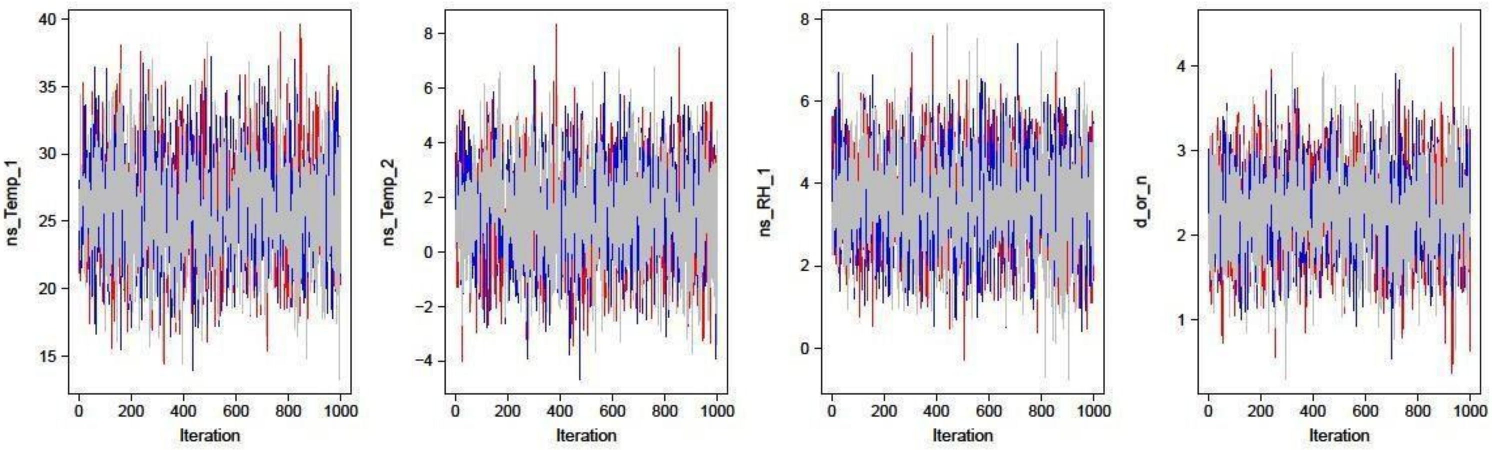

Figure S2f. Trace plots of the parameter values of temperature, relative humidity, and whether the time point was in the daytime or at night (excluding those from warm-up iterations) in the model fitted to male mosquito densities during November 2016 to November 2017 from chain 1 (red lines), chain 2 (blue lines), and chain 3 (grey lines). ns_Temp_k (k=1, 2) indicates the term of a natural cubic spline of temperature; ns_RH_1 indicates the term of a natural cubic spline of relative humidity; d_or_n indicates whether the time point was in the daytime or atnight.

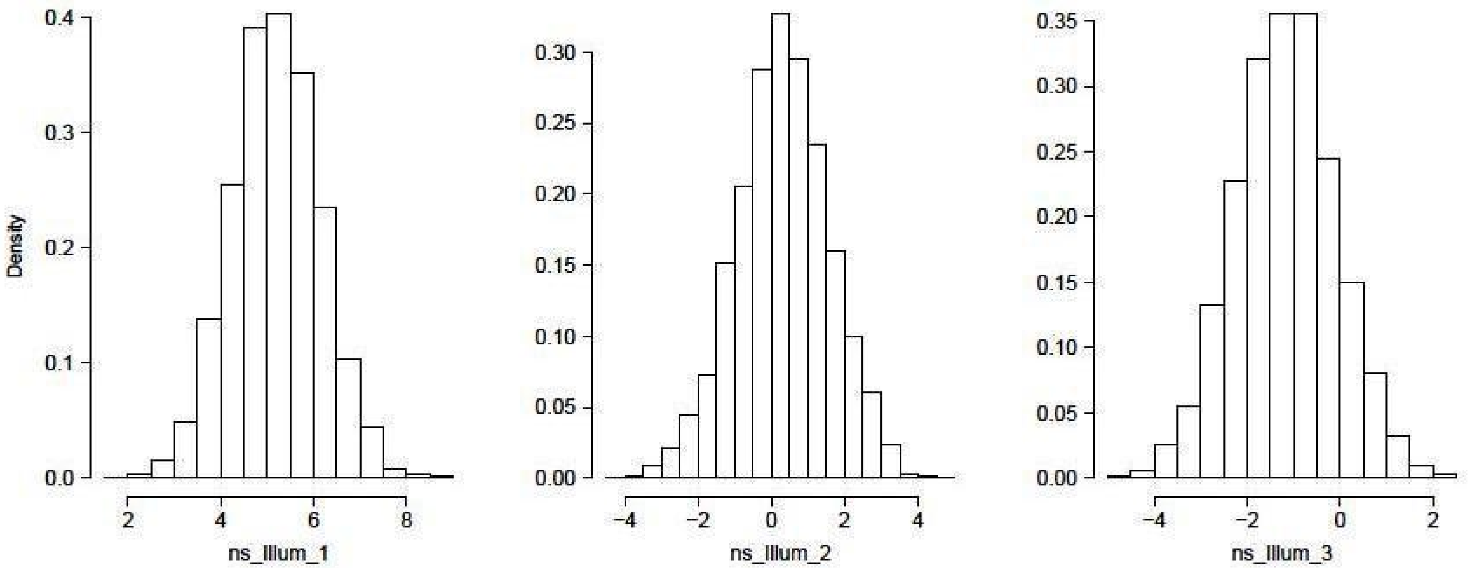

Figure S2g. Histograms of the parameter values of illuminance (excluding those from warm-up iterations) in the model fitted to male mosquito densities during November 2016 to November 2017. ns_Illum_k (k=1, 2, 3) indicates the term of a natural cubic spline of illuminance.

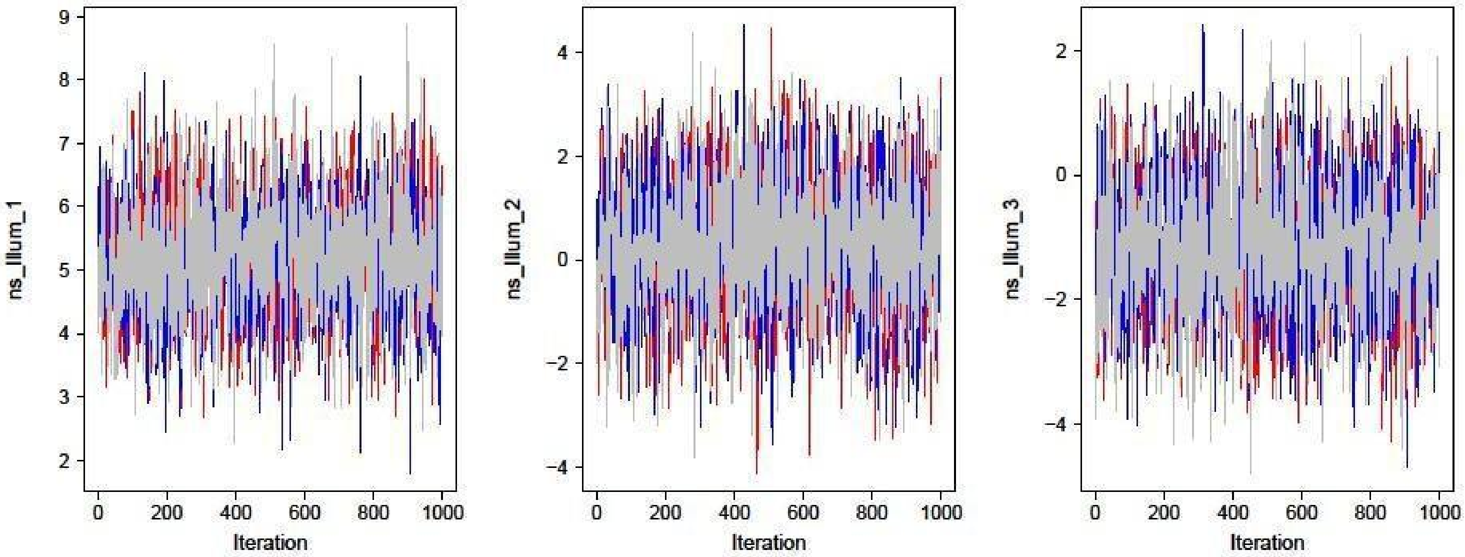

Figure S2h. Trace plots of the parameter values of illuminance (excluding those from warm-up iterations) in the model fitted to male mosquito densities during November 2016 to November 2017 from chain 1 (red lines), chain 2 (blue lines), and chain 3 (grey lines). ns_Illum_k (k=1, 2, 3) indicates the term of a natural cubic spline of illuminance.

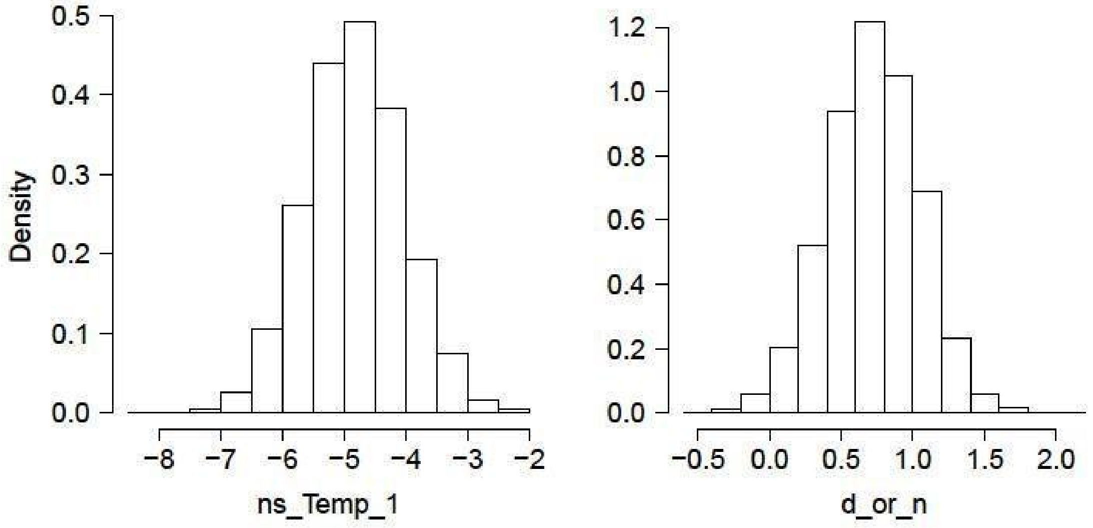

Figure S2i. Histograms of the parameter values of temperature and whether the time point was in the daytime or at night (excluding those from warm-up iterations) in the model fitted to female mosquito densities during June-July 2018. ns_Temp_1 indicates the term of a natural cubic spline of temperature; d_or_n indicates whether the time point was in the daytime or at night.

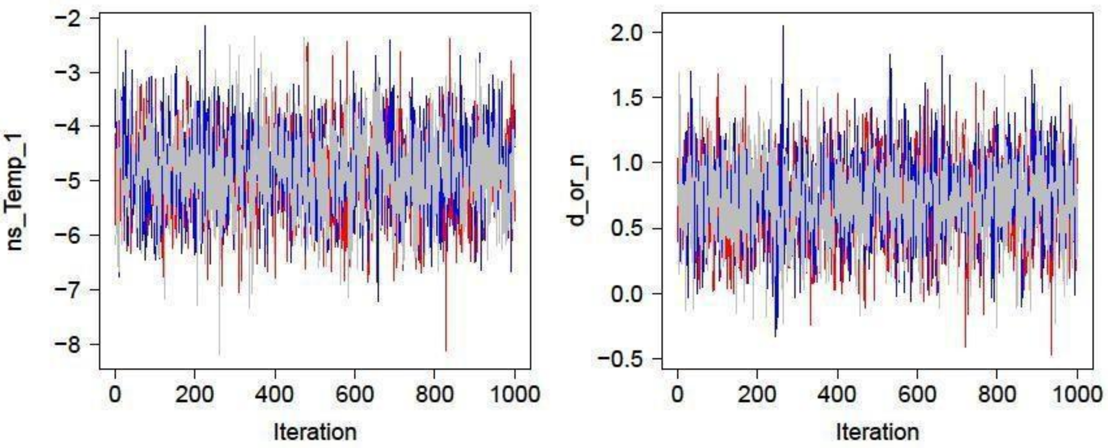

Figure S2j. Trace plots of the parameter values of temperature and whether the time point was in the daytime or at night (excluding those from warm-up iterations) in the model fitted to female mosquito densities during June-July 2018 from chain 1 (red lines), chain 2 (blue lines), and chain 3 (grey lines). ns_Temp_1 indicates the term of a natural cubic spline of temperature; d_or_n indicates whether the time point was in the daytime or at night.

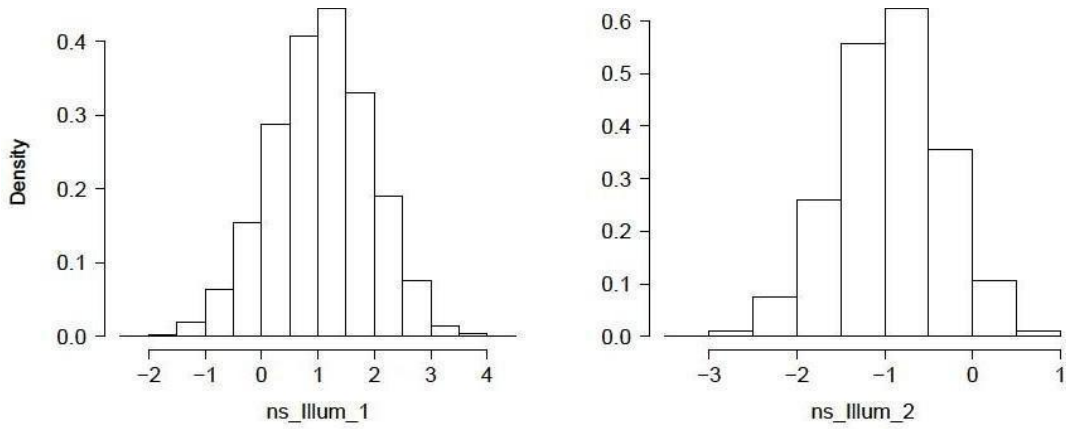

Figure S2k. Histograms of the parameter values of illuminance (excluding those from warm-up iterations) in the model fitted to female mosquito densities during June-July 2018. ns_Illum_k (k=1, 2) indicates the term of a natural cubic spline of illuminance.

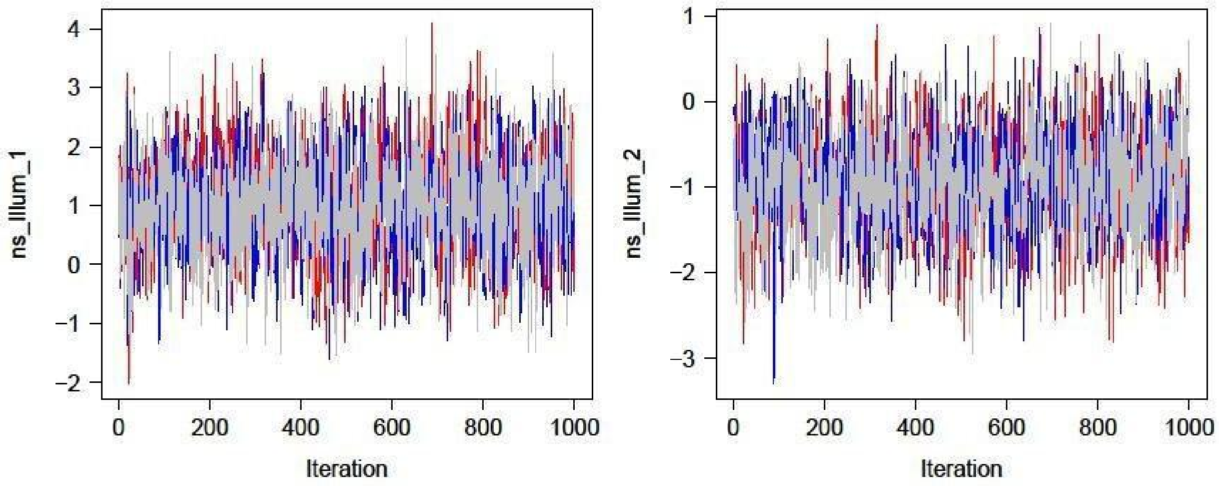

Figure S2l. Trace plots of the parameter values of illuminance (excluding those from warm-up iterations) in the model fitted to female mosquito densities during June-July 2018 from chain 1 (red lines), chain 2 (blue lines), and chain 3 (grey lines). ns_Illum_k (k=1, 2) indicates the term of a natural cubic spline of illuminance.

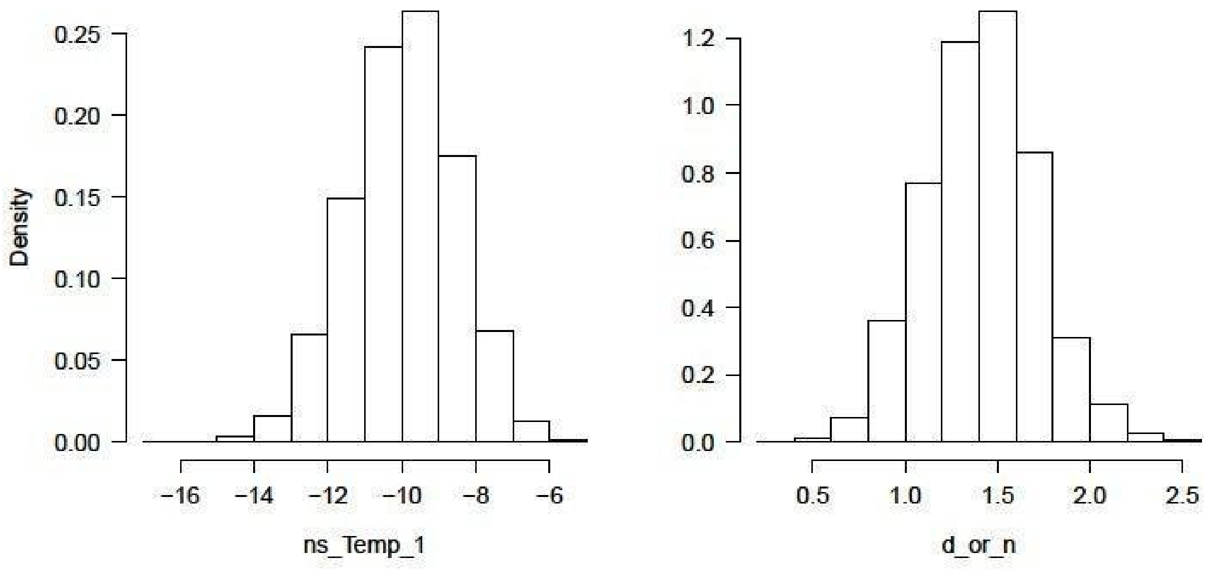

Figure S2m. Histograms of the parameter values of temperature and whether the time point was in the daytime or at night (excluding those from warm-up iterations) in the model fitted to male mosquito densities during June-July 2018. ns_Temp_1 indicates the term of a natural cubic spline of temperature; d_or_n indicates whether the time point was in the daytime or at night.

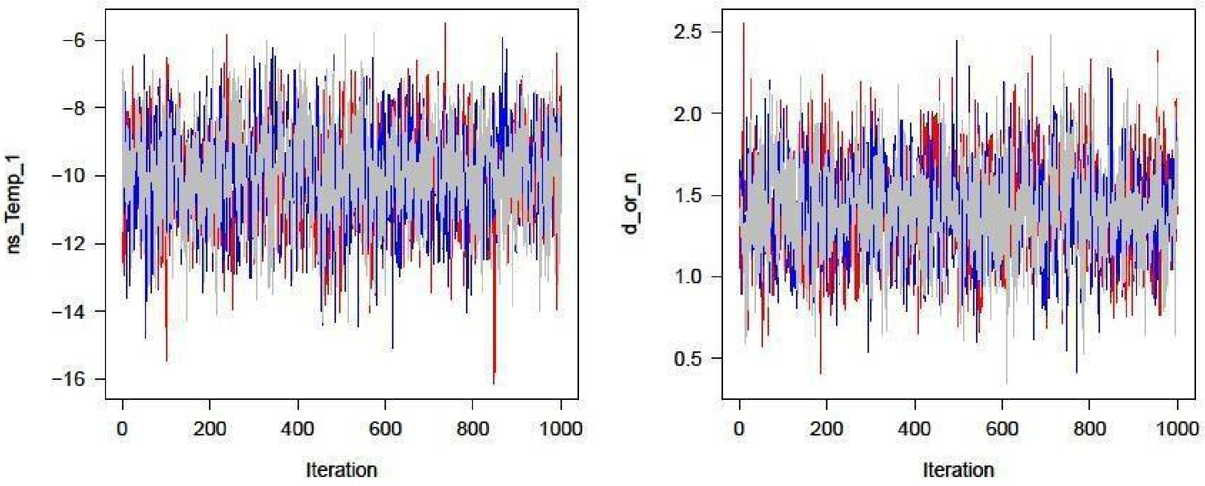

Figure S2n. Trace plots of the parameter values of temperature and whether the time point was in the daytime or at night (excluding those from warm-up iterations) in the model fitted to male mosquito densities during June-July 2018 from chain 1 (red lines), chain 2 (blue lines), and chain 3 (grey lines). ns_Temp_1 indicates the term of a natural cubic spline of temperature; d_or_n indicates whether the time point was in the daytime or at night.

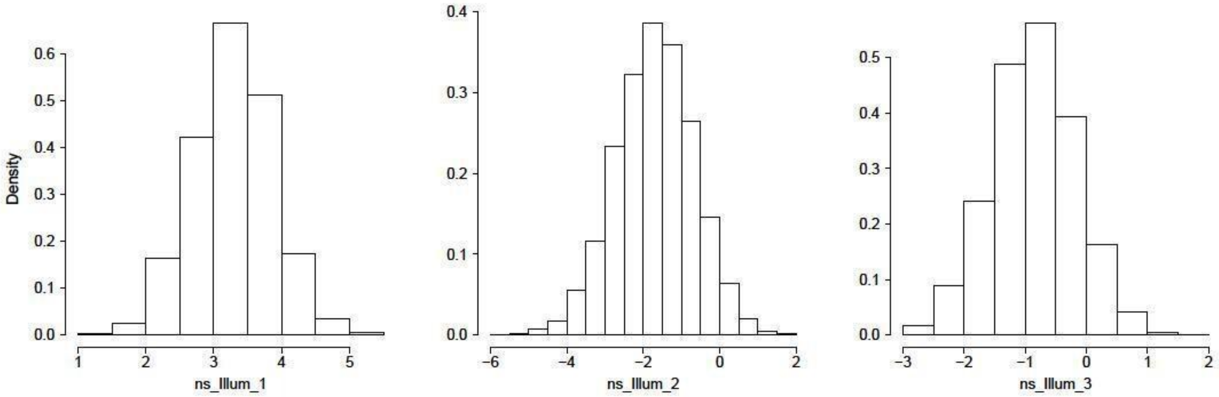

Figure S2o. Histograms of the parameter values of illuminance (excluding those from warm-up iterations) in the model fitted to male mosquito densities during June-July 2018. ns_Illum_k (k=1, 2, 3) indicates the term of a natural cubic spline of illuminance.

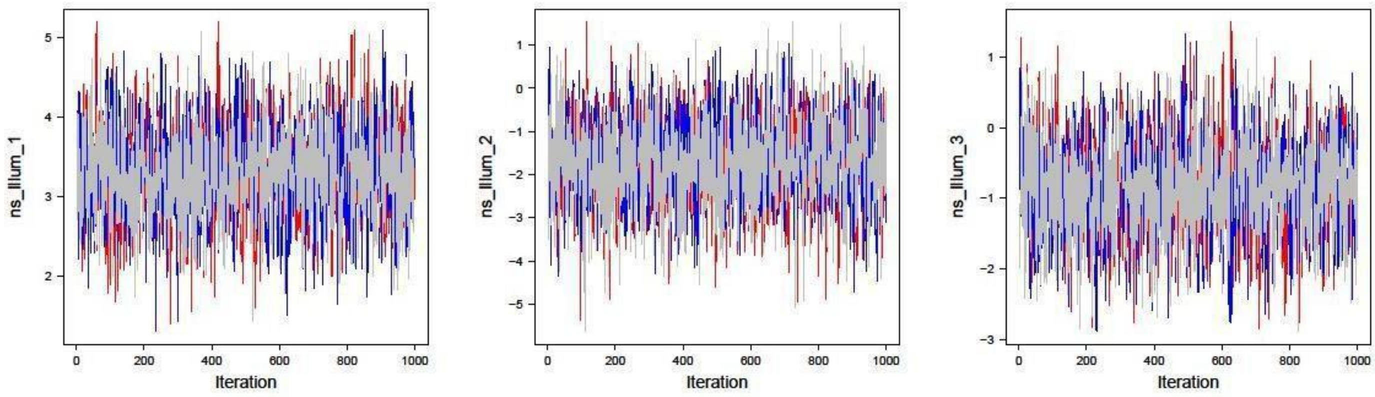

Figure S2p. Trace plots of the parameter values of illuminance (excluding those from warm-up iterations) in the model fitted to male mosquito densities during June-July 2018 from chain 1 (red lines), chain 2 (blue lines), and chain 3 (grey lines). ns_Illum_k (k=1, 2, 3) indicates the term of a natural cubic spline of illuminance.
