# Supplementary material for: A field-based modeling study on ecological characterization of hourly host-seeking behavior and its associated climatic variables in Aedes albopictus
Source: Parasit Vectors. 2019 Oct 14;12:474. doi: 10.1186/s13071-019-3715-1 (PMC6791010; doi:10.1186/s13071-019-3715-1)
Supplement: Supplementary file 12 — Additional file 12: Table S8. Estimated thresholds of temperatures that corresponded to the predicted Ae. albopictus densities which were ≥ 1 per person per hour from multi-month investigations from November 2016 to November 2017. [file 13071_2019_3715_MOESM12_ESM.pdf]

Table S8. Estimated thresholds of temperatures that corresponded to the predicted *Ae. albopictus* densities which were ≥ 1 per person per hour from multi-month investigations from November 2016 to November 2017

Month	Female		Male	
	Low threshold (°C)	High threshold (°C)	Low threshold (°C)	High threshold (°C)
January	None	None	(24.2, 26.8)	None
February	(25.8, 26.3)	27.5	None	None
March	(17.9, 21.8)	None	None	None
April	(16.4, 26.4)	(26.9, 30.5)	(21.7, 28.2)	(28.8, 30.5)
May	(23.4, 26.0)	(26.6, 31.2)	(23.4, 28.2)	(28.8, 31.2)
June	(26.2, 26.4)	(26.6, 36.1)	(26.2, 27.9)	(29.4, 37.1)
July	(25.1, 26.4)	(26.8, 33.9)	(25.1, 28.4)	(28.5, 33.9)
August	None	(26.9, 35.5)	(26.9, 27.9)	(29.3, 35.9)
September	(26.2, 26.5)	(26.6, 37.1)	(26.2, 28.4)	(28.6, 34.4)
October	(25.8, 26.4)	(26.6, 36.8)	(25.8, 28.1)	(28.8, 35.8)
November	(20.2, 26.2)	(26.7, 31.9)	(22.3, 28.3)	(28.6, 31.9)
December	None	None	27.8	None
