# Supplementary material for: A field-based modeling study on ecological characterization of hourly host-seeking behavior and its associated climatic variables in Aedes albopictus
Source: Parasit Vectors. 2019 Oct 14;12:474. doi: 10.1186/s13071-019-3715-1 (PMC6791010; doi:10.1186/s13071-019-3715-1)
Supplement: Supplementary file 13 — Additional file 13: Text S3. Sensitivity analyses. [file 13071_2019_3715_MOESM13_ESM.pdf]

Sensitivity analyses

Methods

Sensitivity analyses were conducted to (i) compare results of the correlation between mosquito hourly densities and climate variables based on the models including and excluding month; (ii) examine the dose-response curves of mosquito hourly densities and climatic variables, including temperature, relative humidity and wind speed based on the models with illuminance instead of *d_or_n*; and (iii) evaluate the associations between mosquito hourly densities and illuminance based on the models with variables including temperature and relative humidity.

Results

Consistent with the results of the models which included month fitted to the data collected during November 2016 to November 2017, the findings based on the models without month also indicated that mosquito hourly densities increased with temperature at first and then decreased (Text S3a). The temperature which corresponded to the highest female mosquito hourly density was 28.5°C (95% credible interval (CrI): 27.7, 29.4°C), which was higher than the estimate based on the model with month (26.5°C (95% CrI: 25.3, 28.1°C)). Mosquito hourly densities increased with illuminance (Text S3a). However, results of the models with month indicated that mosquito hourly densities increased at first and then declined. Mosquito hourly densities increased with relative humidity, while the effect of wind speed on female mosquito density was a little bit complex but was also similar to that estimated from the model with month (Text S3a). Meanwhile, similar results were obtained when fitting model without month to the data collected during June-July 2018, since the data were collected within two months (TextS3b).

The dose-response curves of mosquito hourly densities and climatic variables, including temperature, relative humidity and wind speed estimated from the models with illuminance but without *d_or_n* were similar to those in the main analysis (Text S3c, d). We found that the associations between illuminance and mosquito hourly densities were different estimated from the model including and excluding climatic variables (Text S3e). The temperature, relative humidity could mediate the effects of illuminance on mosquito hourly densities.

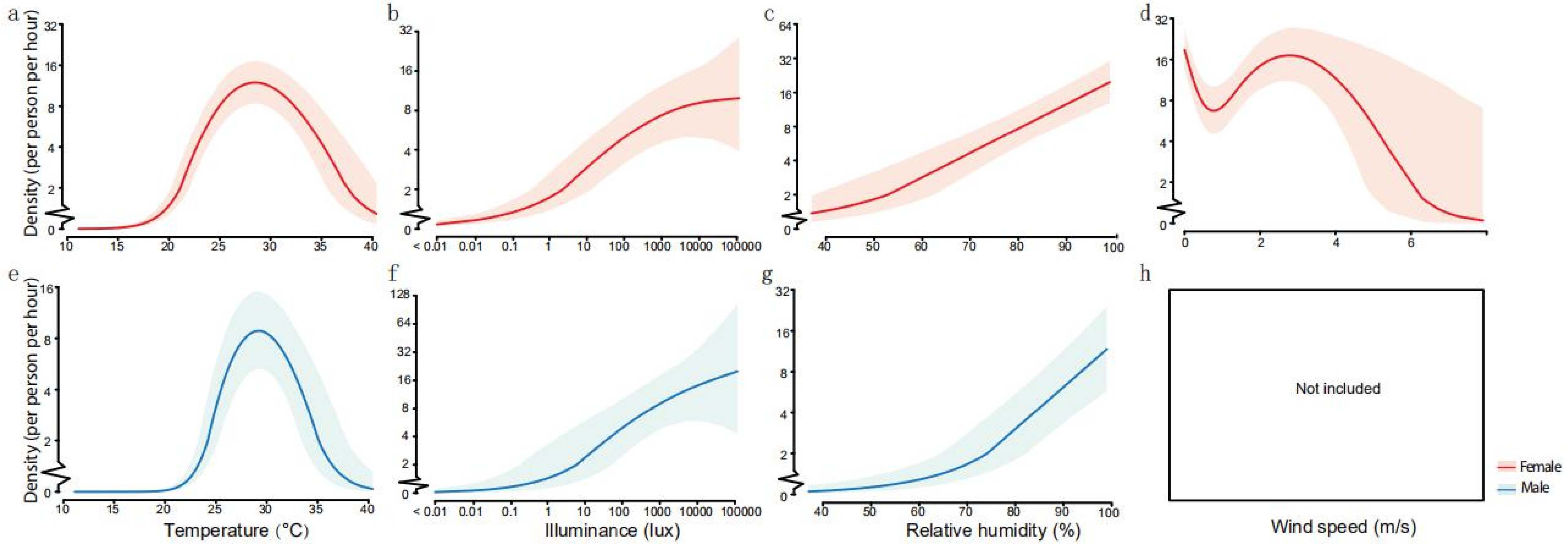

Text S3a. Dose-response relationships between hourly host-seeking activities of *Aedes albopictus* and climatic variables from multi-month investigations based on the models excluding month. The solid lines represent the predicted values of *Ae. albopictus* hourly densities by temperature, illuminance, relative humidity and wind speed, assuming that the other continuous covariates were equal to their medians and that the time point was at 18:00 h in June. The shaded areas represent the 95% credible intervals of predicted hourly densities. **a-d** Dose-response relationships between hourly host- seeking activities of female *Ae. albopictus* and temperature, illuminance, relative humidity and wind speed in multi-month investigations from November 2016 to November 2017. **e-h** Dose-response relationships between hourly host-seeking activities of male *Ae. albopictus* and temperature, illuminance, relative humidity and wind speed in multi-month investigations from November 2016 to November 2017.

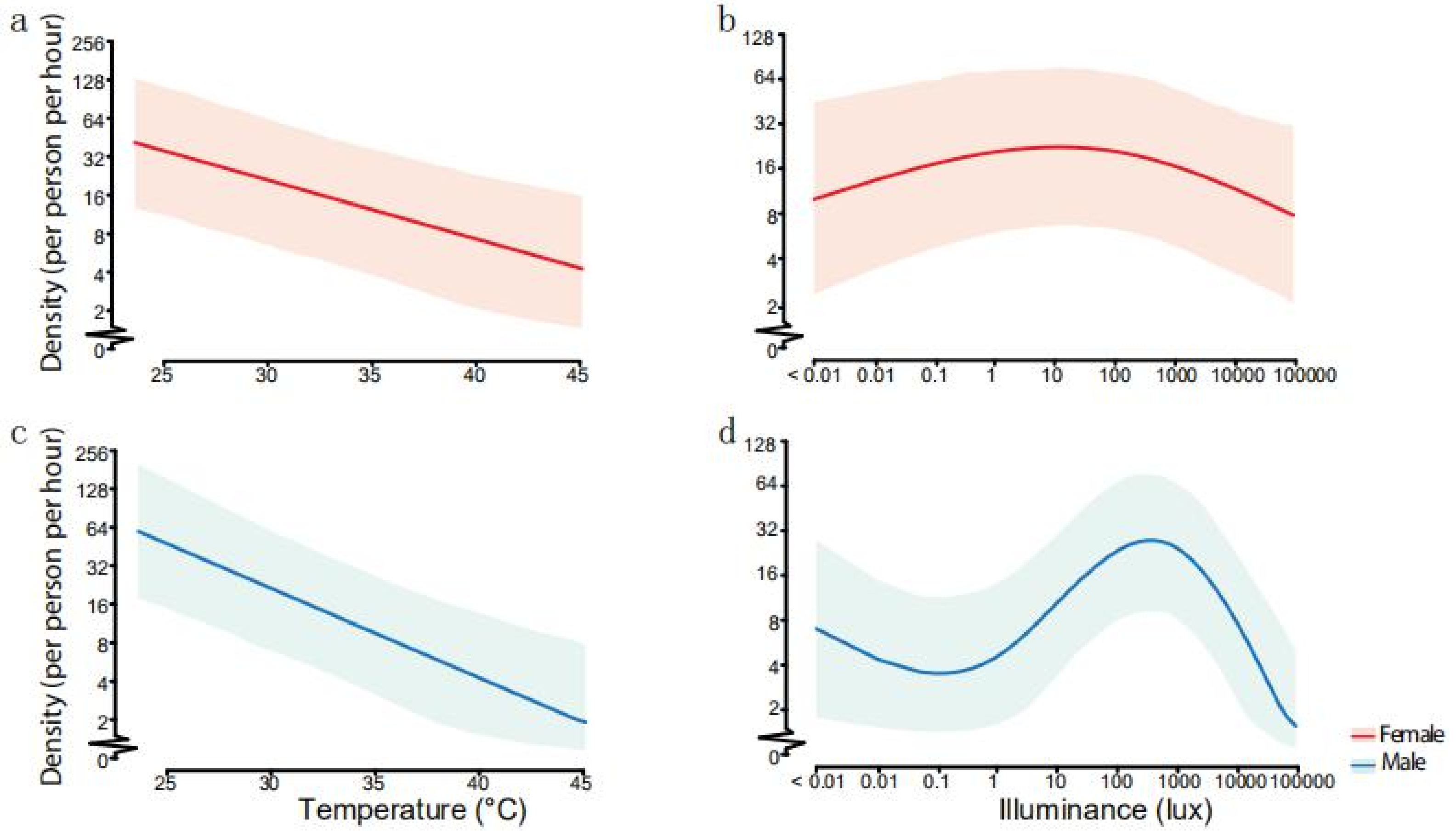

Text S3b. Dose-response relationships between hourly host-seeking activities of *Ae. albopictus* and climatic variables from multi-site investigations based on the models excluding month. The solid lines represent the predicted values of *Ae. albopictus* hourly densities by temperature and illuminance, assuming that the other continuous covariates were equal to their medians and that the time point was at 18:00 h in June. **a, b** Dose-response relationships between hourly host-seeking activities of female *Ae. albopictus* and temperature, illuminance, in multi-site investigations during June-July 2018. **c, d** Dose-response relationships between hourly host-seeking activities of male *Ae. albopictus* and temperature, illuminance, in multi-site investigations during June-July 2018.

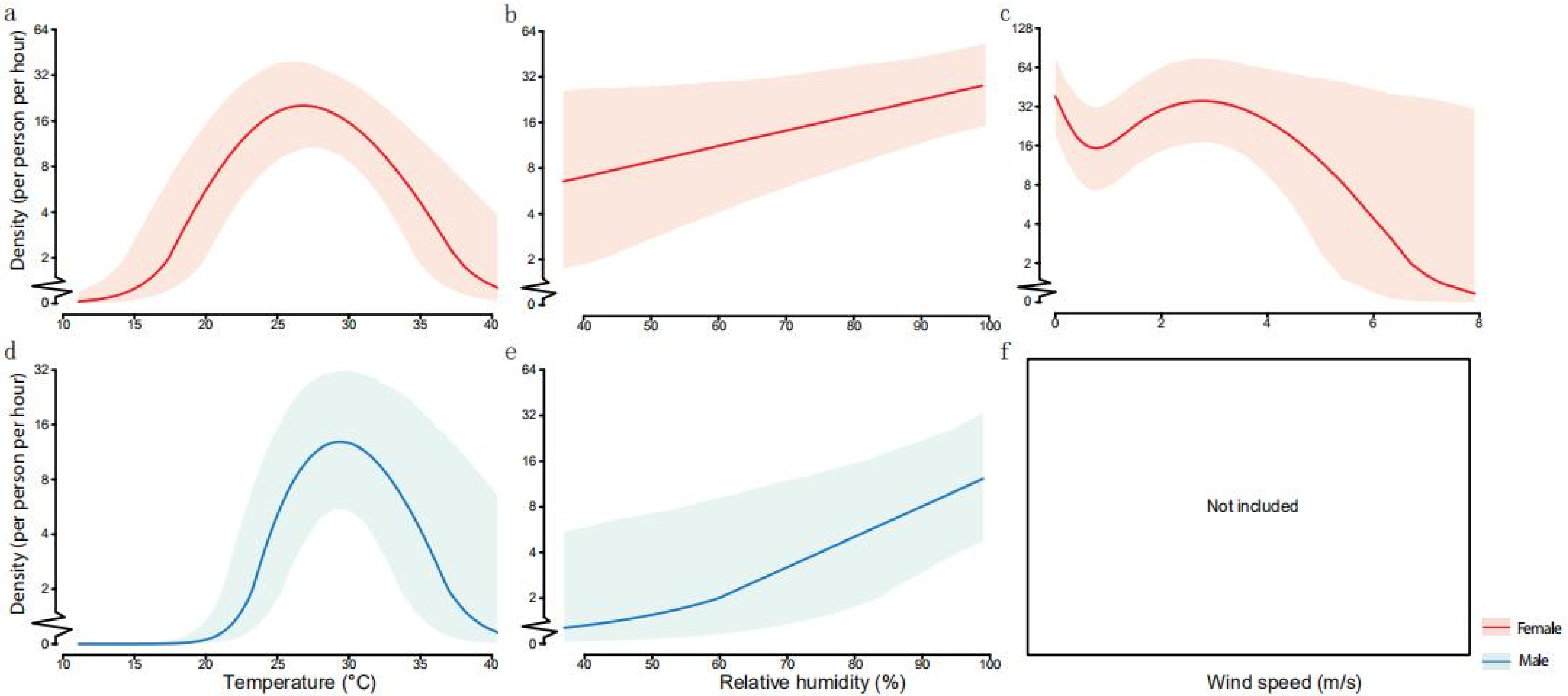

Text S3c. Dose-response relationships between hourly host-seeking activities of *Ae. albopictus* and climatic variables estimated from the models with illuminance from multi-month investigations. The solid lines represent the predicted values of *Ae. albopictus* hourly densities by climatic variates, assuming that the other continuous covariates were equal to their medians and that the time point was at 18:00 h in June. The shaded areas represent the 95% credible intervals of predicted hourly densities. **a-c** Dose-response relationships between hourly host-seeking activities of female *Ae. albopictus* and temperature, relative humidity and wind speed in multi-month investigations from November 2016 to November 2017. **d-f** Dose-response relationships between hourly host-seeking activities of male *Ae. albopictus* and temperature, relative humidity, and wind speed in multi-month investigations from November 2016 to November 2017.

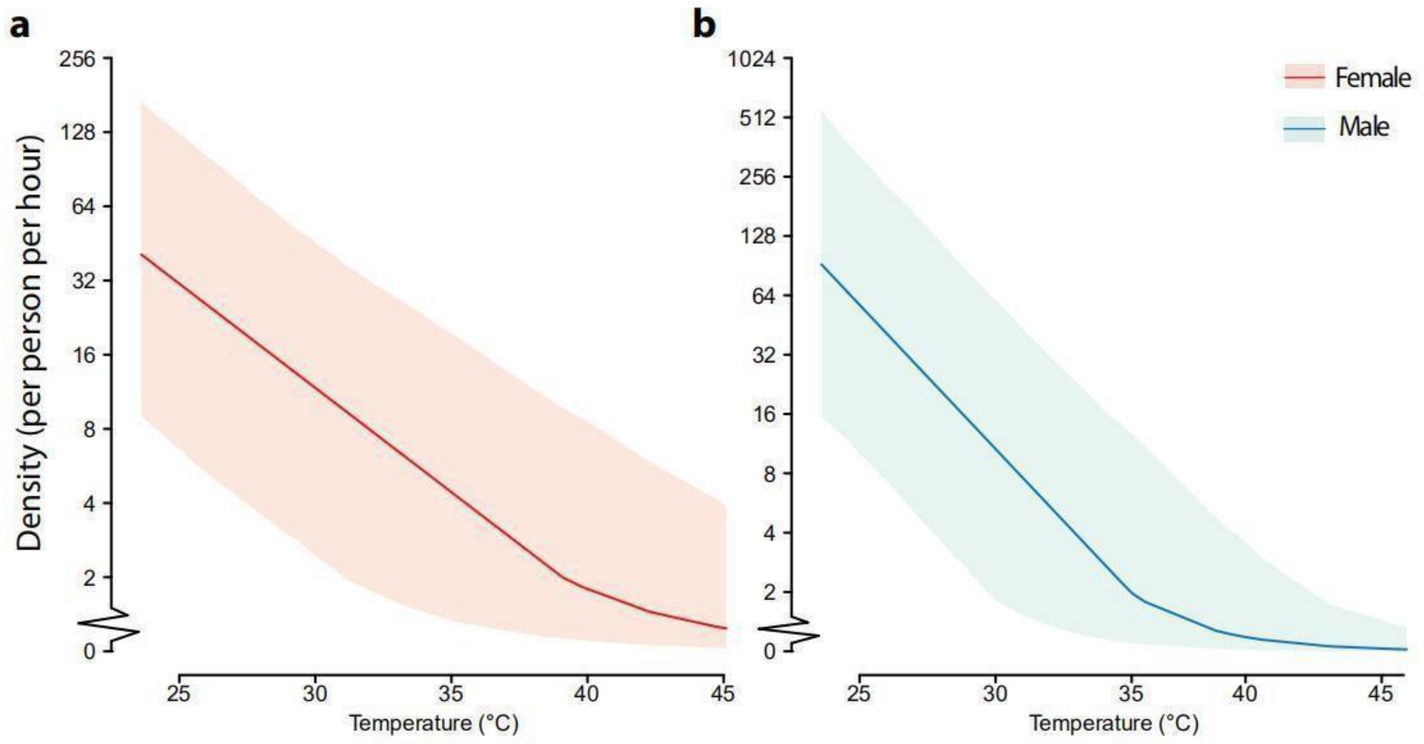

Text S3d. Dose-response relationships between hourly host-seeking activities of *Ae. albopictus* and temperature estimated from the models with illuminance from multi-site investigations. The solid lines represent the predicted values of *Ae. albopictus* hourly densities by temperature, assuming that the other continuous covariates were equal to their medians and that the time point was at 18:00 h in June. The shaded areas represent the 95% credible intervals of predicted hourly densities. **a** Dose-response relationships between hourly host-seeking activities of female *Ae. albopictus* and temperature in multi- site investigations during June-July 2018. **b** Dose-response relationships between hourly host-seeking activities of male *Ae. albopictus* and temperature in multi-site investigations during June-July2018.

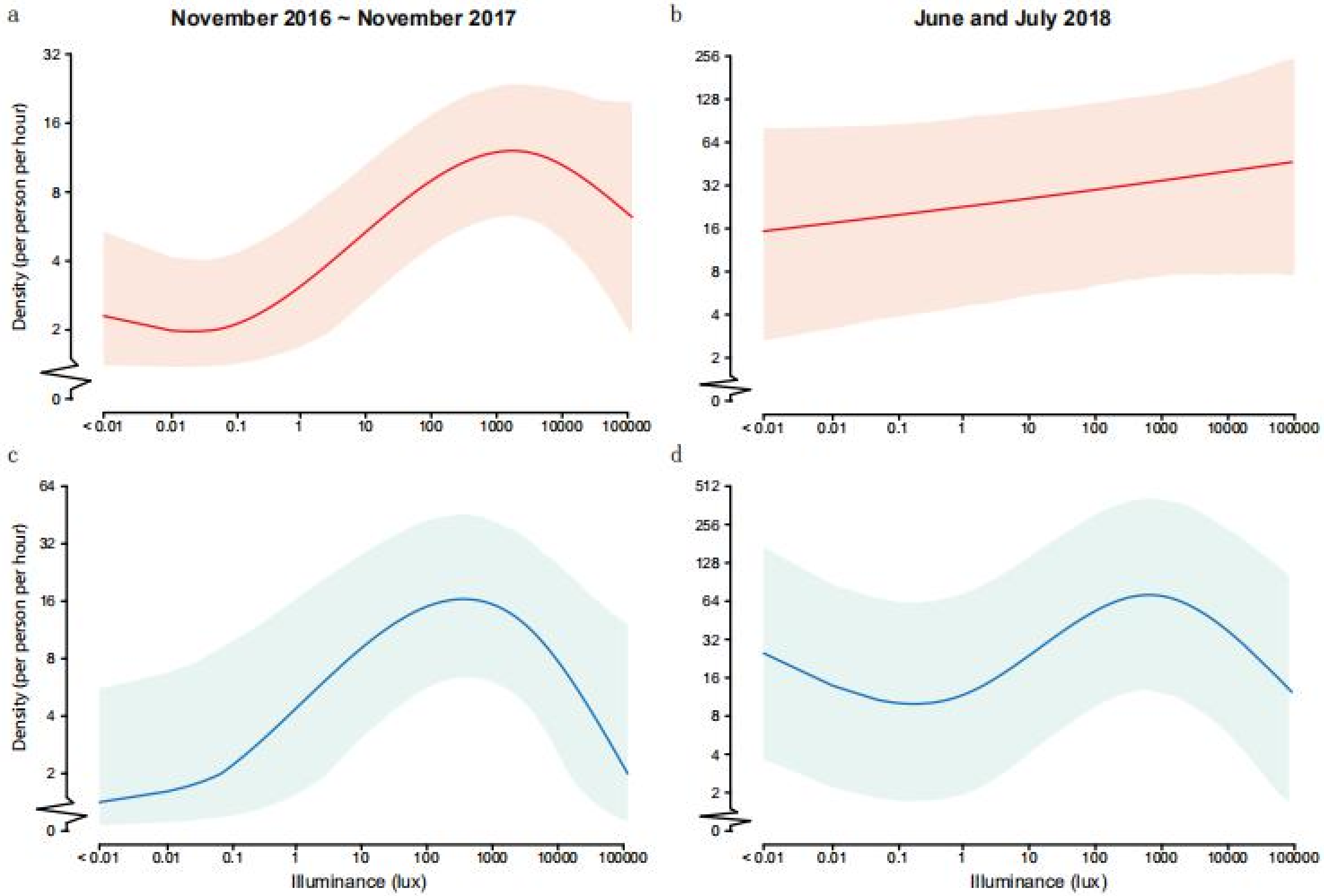

Text S3e. Dose-response relationships between mosquito hourly densities and illuminance based on the models with climatic variables. The solid lines represent the predicted values of *Ae. albopictus* hourly densities by illuminance, assuming that the other continuous covariates were equal to their medians and that the time point was at 18:00 h in June. The shaded areas represent the 95% credible intervals of predicted hourly densities. **a, c** Dose-response relationships between hourly host-seeking activities of female (a) and male (c) *Ae. albopictus* and illuminance in multi-month investigations from November 2016 to November 2017. **b, d** Dose-response relationships between hourly host-seeking activities of female (b) and male (d) *Ae. albopictus* and illuminance in multi-site investigations during June-July 2018.
